# Silencing of Iron and Heme-Related Genes Revealed a Paramount Role of Iron in the Physiology of the Hematophagous Vector *Rhodnius prolixus*

**DOI:** 10.3389/fgene.2018.00019

**Published:** 2018-02-02

**Authors:** Ana B. Walter-Nuno, Mabel L. Taracena, Rafael D. Mesquita, Pedro L. Oliveira, Gabriela O. Paiva-Silva

**Affiliations:** ^1^Instituto de Bioquímica Médica Leopoldo de Meis, Universidade Federal do Rio de Janeiro, Rio de Janeiro, Brazil; ^2^Instituto Nacional de Ciência e Tecnologia em Entomologia Molecular, Rio de Janeiro, Brazil; ^3^Departamento de Bioquímica, Instituto de Química, Universidade Federal do Rio de Janeiro, Rio de Janeiro, Brazil

**Keywords:** iron, heme, hematophagy, insect, oxidative stress, genome, gene silencing, *Rhodnius prolixus*

## Abstract

Iron is an essential element for most organisms However, free iron and heme, its complex with protoporphyrin IX, can be extremely cytotoxic, due to the production of reactive oxygen species, eventually leading to oxidative stress. Thus, eukaryotic cells control iron availability by regulating its transport, storage and excretion as well as the biosynthesis and degradation of heme. In the genome of *Rhodnius prolixus*, the vector of Chagas disease, we identified 36 genes related to iron and heme metabolism We performed a comprehensive analysis of these genes, including identification of homologous genes described in other insect genomes. We observed that blood-meal modulates the expression of ferritin, Iron Responsive protein (IRP), Heme Oxygenase (HO) and the heme exporter Feline Leukemia Virus C Receptor (FLVCR), components of major pathways involved in the regulation of iron and heme metabolism, particularly in the posterior midgut (PM), where an intense release of free heme occurs during the course of digestion. Knockdown of these genes impacted the survival of nymphs and adults, as well as molting, oogenesis and embryogenesis at different rates and time-courses. The silencing of FLVCR caused the highest levels of mortality in nymphs and adults and reduced nymph molting. The oogenesis was mildly affected by the diminished expression of all of the genes whereas embryogenesis was dramatically impaired by the knockdown of ferritin expression. Furthermore, an intense production of ROS in the midgut of blood-fed insects occurs when the expression of ferritin, but not HO, was inhibited. In this manner, the degradation of dietary heme inside the enterocytes may represent an oxidative challenge that is counteracted by ferritins, conferring to this protein a major antioxidant role. Taken together these results demonstrate that the regulation of iron and heme metabolism is of paramount importance for *R. prolixus* physiology and imbalances in the levels of these key proteins after a blood- meal can be extremely deleterious to the insects in their various stages of development.

## Introduction

Due to its ability to change its oxidation state, iron as such or as heme, its complex with protoporphyrin IX, participates in redox reactions that are required by essential physiological processes such as cell signaling and energy metabolism (Ponka, 1999; Mense and Zhang, 2006; Muckenthaler et al., 2008). However, free iron or heme can be cytotoxic (Aft and Mueller, 1983, 1984; Gutteridge and Smith, 1988; Vincent, 1989; Schmitt et al., 1993; Ryter and Tyrrell, 2000). In the presence of oxygen, by means of the Fenton reaction, iron can produce reactive oxygen species, leading to cellular redox imbalance and an oxidative stress (Papanikolaou and Pantopoulos, 2005).

The control of iron and heme homeostasis is particularly critical for hematophagous insects, many of which are vectors of major human life-threatening or life-long diseases caused by the pathogens that are taken along with the blood meal. High amounts of iron and heme are released during the digestion of host blood. Thus, in the course of evolution, several mechanisms to avoid iron and heme overload and oxidative damage have appeared in these organisms, contributing to their adaptation to hematophagy (Graca-Souza et al., 2006). As a first line of defense against heme toxicity, some blood-sucking arthropods sterically isolate heme molecules, reducing their reactivity. In the hemipteran *Rhodnius prolixus*, most of free heme molecules derived from hemoglobin digestion are aggregated as hemozoin, which results in the reduction of free radical formation in the midgut lumen (Oliveira et al., 1999, 2002). Likewise, the cattle tick *Rhipicephalus microplus* accumulates the dietary heme in specialized membrane-bound organelles called hemosome, found in the midgut digestive cells (Lara et al., 2003).

Another mechanism to prevent iron and heme-induced damages is the expression of proteins that control iron availability by regulating its absorption, transport, storage and excretion as well as the biosynthesis and degradation of heme (Lane et al., 2015). Although some of these genes have already been described in insects, their role in the general physiology and in the adaptation of blood-sucking insects to the hematophagic habit are poorly understood (Mandilaras et al., 2013; Tang and Zhou, 2013b).

Here, we identified genes related to iron and heme metabolism in the genome of *R. prolixus*, one of the main vectors of Chagas disease (Mesquita et al., 2015) and evaluated the relevance of a selected group of these genes in the physiology of the insect. The expression levels of these genes in each of the midgut compartments were also measured and their role in the adaptation *of R. prolixus* to hematophagy was discussed.

## Materials and Methods

### Gene Sequence Analyses

Gene sequence analyses were based on the genome assembly (Rproc1 version) and gene predictions (VectorBase 1.3 version) performed by Mesquita et al. (2015) and deposited in VectorBase database^1^. Uncompleted or fragmented genes were re-predicted using exonerate software version 2.2^2^ (Slater and Birney, 2005) based on insect orthologous proteins sequences deposited in NCBI databases^3^. The re-prediction results were manually curated and edited when needed. Re-predicted sequences are available as Supplementary Material.

### Analysis of Protein Domains

Transmembrane regions were predicted using TMHMM version 2.0^4^ (Krogh et al., 2001). Signal peptide cleavage sites were predicted using SignalP software version 4.1^5^. Mitochondria targeting sequences were predicted using TargetP version 1.1^6^ (Emanuelsson et al., 2007; Nielsen, 2017) and MITOPROT^7^ (Claros and Vincens, 1996), while GPI-modification anchors were searched using PredGPI version 3.0^8^ (Pierleoni et al., 2008).

### Iron Responsive Elements (IREs) Prediction

Iron responsive elements stem-loop structures were screened in the upstream regions (1200 bp) of ferritin genes using the Search for IREs (SIRE) server version 2.0^9^ (Campillos et al., 2010).

### Multiple Sequence Alignments

Multiple alignments of protein sequences were performed using ClustalW with the default configuration (Thompson et al., 2002). Multiple sequences alignment figures were created using BioEdit software (Hall, 1999).

### Phylogenetic Trees

Unrooted trees were calculated by the maximum-likelihood method with RAxML version 8 (Stamatakis, 2014) using PROTCAT+JTT models and bootstrap support with 500 replicates. FigTree software version 1.4 was used to draw the trees^10^.

### Insect Rearing

Insects were taken from a colony of *R. prolixus* maintained at 28°C and 80–90% relative humidity under a photoperiod of 12 h of light/12 h of dark. The animals used in this work were mated females fed on rabbit blood at 3-week intervals. *R prolixus* female injected with dsRNA were kept in individual vials maintained under the same conditions. In RNAi experiments, nymphs (first stage, N1) were artificially fed on heparinized blood supplemented with dsRNA (1 μg/μL) through a latex membrane stretched across the bottom of a water-jacketed glass feeder apparatus kept at 37°C.

### Ethics Statement

All animal care and experimental protocols were conducted following the guidelines of the institutional care and use committee (Committee for Evaluation of Animal Use for Research from the Federal University of Rio de Janeiro), which are based on the National Institutes of Health Guide for the Care and Use of Laboratory Animals (ISBN0-309-05377-3). The protocols were approved by the Committee for Evaluation of Animal Use for Research (CAUAP) from the Federal University of Rio de Janeiro, under registry number 115/13. Technicians dedicated to the animal facility at the Institute of Medical Biochemistry (Federal University of Rio de Janeiro) carried out all aspects related to rabbit husbandry under strict guidelines to ensure careful and consistent handling of the animals.

### Tissue Isolation and RNA Extraction

Anterior and posterior midguts (PMs) from starved and blood-fed females were dissected at different days after blood ingestion. Total RNA was extracted from individual tissues or pools of 3–5 midguts using TRIzol reagent (Invitrogen, Carlsbad, CA, United States) according to the manufacturer’s instructions. RNA concentrations were determined spectrophotometrically at 260 nm on a Nanodrop 1000 spectrophotometer 3.7 (Thermo Fisher Scientific). Following treatment with RNase-free DNaseI (Fermentas International Inc., Burlington, Canada), 1 μg of RNA was used for cDNA synthesis with a high capacity cDNA reverse transcription kit (Applied Biosystems, Foster City, CA, United States) and random hexamers according to the manufacturer’s instructions.

### dsRNA Synthesis and Gene Silencing Assays

Fragments of 300–400 bp were amplified by PCR using cDNA from midgut epithelia from blood-fed females (48 h after feeding) produced as described above. The following conditions were used for amplification: one cycle for 5 min at 95°C, followed by 40 cycles of 30 s a 95°C, 30 s at 63°C, and 1 min at 72°C, with a final step of 10 min at 72°C. The oligonucleotides used for amplification of templates for dsRNA synthesis are listed at Supplementary Table 1. These primers contained a T7 polymerase binding sequence, required for dsRNA synthesis. Amplified cDNAs were used as a template for dsRNA synthesis using a MEGAScript RNAi kit (Ambion Inc., Austin, TX, United States) according to the manufacturer’s instructions. The maltose-binding protein (MAL) gene from *Escherichia coli* (gene identifier 7129408) in a pBlueScript KS (Stratagene) was amplified by PCR using T7 minimal promoter primers under the following conditions: one cycle for 10 min at 95°C, followed by 40 cycles of 15 s at 95°C, 15 s at 45°C, and 45 s at 72°C, with a final step of 10 min at 72°C. The PCR product produced was used as a template for Mal dsRNA synthesis used as a control in the silencing assays. Following *in vitro* synthesis, all dsRNAs were purified according to the manufacturer’s instructions. RNAi experiments were performed by injection of 1 μL of dsRNA (1 μg/μL) from the iron or heme–related genes HCH ferritin (RPRC009256), IRP (RPRC001246), HO (RPRC006832), and FLVCR (RPRC015407) into the thoraxes of starved adult females. Insects were fed with blood 48 h after injection and midgut epithelia were dissected at different times after feeding for RNA extraction as described above.

### Quantitative RT-PCR Assays

Quantitative PCR was performed in a 7500 real-time PCR system (Applied Biosystems, CA, United States) using SYBR Green PCR Master Mix (Applied Biosystems, CA, United States) under the following conditions: one cycle for 10 min at 95°C, followed by forty cycles of 15 s at 95°C and 45 s at 60°C. PCR amplification was performed using the oligonucleotides specified in Supplementary Table 2. *R. prolixus* elongation factor 1 gene (RPRC007684) expression was used as an internal control for normalization (Majerowicz et al., 2011). *C*t values were calculated from *C*t (cycle threshold) values obtained from quantitative RT-PCR and were used to calculate relative expression and perform statistical analysis (Livak and Schmittgen, 2001). The relative expression values based on 2^-ΔΔ*C*_T_^ were used in silenced genes analyses and 2^-Δ*C*_T_^ for gene expression to allow comparison between anterior and PMs. For evaluation of iron/heme-related gene expression in AM and PM, three independent experiments were performed. Each one analyzed 4–5 different individual samples (pools of 3–5 midguts each).

### Analysis of Survival, Oviposition and Egg Viability

Two days prior to the blood meal, adult females were injected with 1 μL of dsRNA (1 μg/μL) into the thoracic cavity. dsRNA-injected and fed *R. prolixus* females (as described above) were kept in individual vials under controlled temperature and humidity conditions. The mortality and oviposition were followed for the 21 days. The eggs laid were collected daily and kept at 28°C and 80% humidity until hatching. Egg eclosion and nymphs molting (N1 to N2) were monitored daily for 30 days.

### ROS Detection in the Midgut

Starved females were injected with 1 μg of Fer dsRNA, HO dsRNA or Mal dsRNA (control), 48 h before blood feeding. Two days after the meal, wings, legs and dorsal plaques were removed by dissection and the insect hemocoel was filled with 50 μM of the oxidant-sensitive fluorophore dihydroethidium (hydroethidine; DHE; Invitrogen) diluted in Leibovitz-15 media supplemented with 5% fetal bovine serum. The samples were incubated in the dark at 28°C for 30 min. After that, the incubation media was removed. The midguts were washed with 0.15 M NaCl and immediately transferred to a glass slide for fluorescence microscopy analysis. Quantitative evaluation of the oxidized-DHE fluorescence levels was performed by acquiring images under identical conditions using a 20× objective and 80 ms of exposure, to allow comparison of different samples. The images were acquired in a Zeiss Observer.Z1 with a Zeiss Axio Cam MrM Zeiss and the data were analyzed using AxioVision version 4.8 software. The filter set (excitation BP 546/12 nm; beam splitter FT 580 nm; emission LP 590 nm) was used for DHE labeling.

### Statistical Analyses

Statistical analysis was performed using a one-way or two-way analysis of variance followed by Tukey’s multiple comparisons *post hoc* test (GraphPad Prism software, San Diego, CA, United States). Details on the sample sizes, appropriate test used and results of the statistical analyzes are indicated in the respective legend figures. All experiments were carried out independently at least three times.

## Results

In the analysis of the genome of *R. prolixus* we identified 36 gene homologs to known to be iron and heme-related proteins previously found in other organisms. These genes were classified into three groups according to their functions: iron binding and storage, iron transmembrane traffic and heme metabolism.

### Iron Binding and Storage Genes

#### Ferritins and Iron Responsive Binding Proteins

Ferritins are ubiquitous iron binding proteins involved in iron storage and transport. In vertebrates, they are also considered to provide antioxidant protection due to the high toxicity of free iron (Andrews and Schmidt, 2007; Lane et al., 2015). Ferritins are multimeric proteins composed of two types of subunits that, in insects, are named heavy and light chain homologs (HCH and LCH, respectively). HCH are characterized by conserved amino acid residues that compose the ferroxidase center responsible for the oxidation of Fe^2+^ whereas LCH is involved in iron nucleation (Andrews and Schmidt, 2007; Mandilaras et al., 2013; Tang and Zhou, 2013a). In contrast to vertebrate and plant ferritins that are cytosolic proteins, most insect ferritin genes display a typical signal peptide and are secreted proteins (Pham and Winzerling, 2010; Mandilaras et al., 2013; Tang and Zhou, 2013a).

**FIGURE 1:**
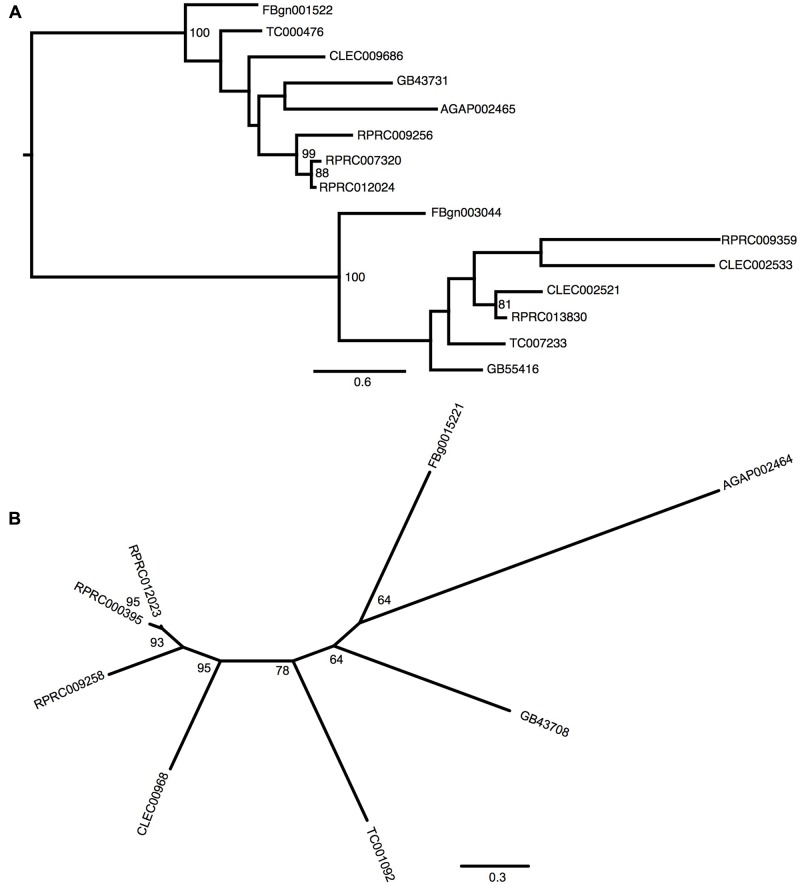
Phylogenetic analysis of ferritins orthologs. Maximum-likelihood trees of ferritins HCH **(A)** and LCH **(B)** orthologs. Numbers on the branches are bootstrap support values from 500 replicates. Only numbers 50% or higher are shown. The sequence codes used were *Rhodnius prolixus* (RPRC), *Drosophila melanogaster* (FBg), *Tribolium castaneum* (TC), *Cimex lectularius* (CLEC), *Apis mellifera* (GB) and *Anopheles gambiae* (AGAP).

**FIGURE 2:**
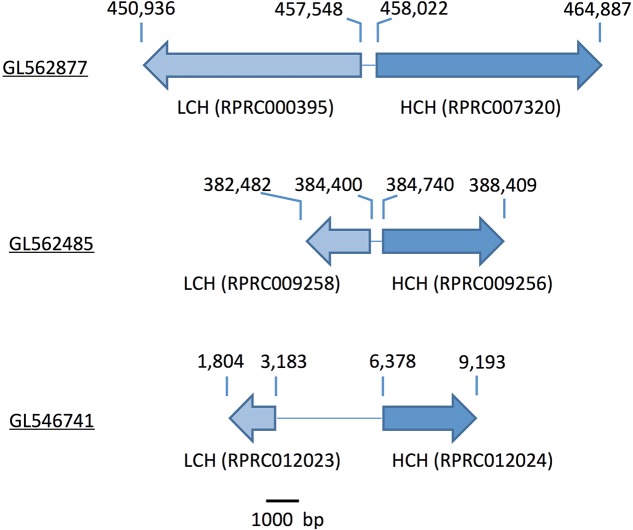
Scheme of the genomic distribution of the secreted HCH and LCH ferritins subunits in the genome of *R. prolixus*. Scaffold numbers are underlined. The numbers above the arrows indicate the position within the scaffold. Ferritin subunits (gene codes) are indicated below the arrows.

Gene analysis of insects of different orders such as Diptera (*Drosophila melanogaster* and *Anopheles gambiae*), Hymenoptera (*Apis Mellifera*), Coleoptera (*Tribolium castaneum*) and Hemiptera (*Cimex lectularius*) reveals that all of them have one pair of genes encoding for secreted ferritin polypeptides (one gene for each subunit). Remarkably, the *R. prolixus* genome comprises three pairs of genes that encode for different secreted HCH and LCH subunits (RPRC007320, RPRC009256, RPRC012024 for HCH and RPRC000395, RPRC009258, RPRC012023 for LCH) (Supplementary Figures 1A,B). Secreted ferritin HCHs are very similar in aminoacid sequence (Supplementary Figure 1A) and are phylogenetic related (**Figure 1A**). The same is observed for LCH subunits (**Figure 1B** and Supplementary Figure 1B) These genes are localized in different regions of the genome, clustered in pairs (one HCH and one LCH) in a head-to-head position, as described for other insects (Dunkov and Georgieva, 2006) (**Figure 2**).

In addition to the secreted HCH ferritins, *R. prolixus* transcribes an HCH subunit that does not present a signal peptide, which is consistent with a cytosolic protein (RPRC013830) and a putative mitochondrial subunit RPRC009359 that has a mitochondria-addressing peptide. In both subunits, the amino acid residues involved in the ferroxidase activity are conserved as well (Supplementary Figure 1A). Phylogenetic analysis separated secreted and non-secreted ferritin HCHs into two groups (**Figure 1A**). Cytosolic and mitochondria subunits are grouped with non-secreted HCH insect ferritins. Futhermore, both cytosolic and mitochondrial genes have closelly-related orthologs in the *C. lectularius*, that also belongs to the order Hemiptera. No cytosolic or mitochondrial LCH genes were found in *R. prolixus* genome.

Besides regulation of transcription, ferritins and other iron/heme-related proteins such as the aminolevulinic acid synthase (ALAS), responsible for the first step of heme biosynthesis, are also regulated post-transcriptionally according to intracellular iron levels (Pantopoulos, 2004; Wilkinson and Pantopoulos, 2014). Under conditions of low iron availability, IRP (Iron Responsive Element Binding Protein) binds to a stem-loop structure found in the 5′ untranslated regions of the mRNAs, named iron-responsive element (IRE), sterically blocking mRNA translation of these proteins. When an iron atom binds to IRP due to increased iron levels, the protein reduces its affinity to the IRE (Pantopoulos, 2004; Wilkinson and Pantopoulos, 2014). We identified canonical IREs in the 5′UTR of all secreted HCH mRNAs (Supplementary Figure 2). Neither the LCH ferritins nor the cytosolic and mitochondrial HCH contain IREs in the 5′UTRs in their mRNAs.

**FIGURE 3:**
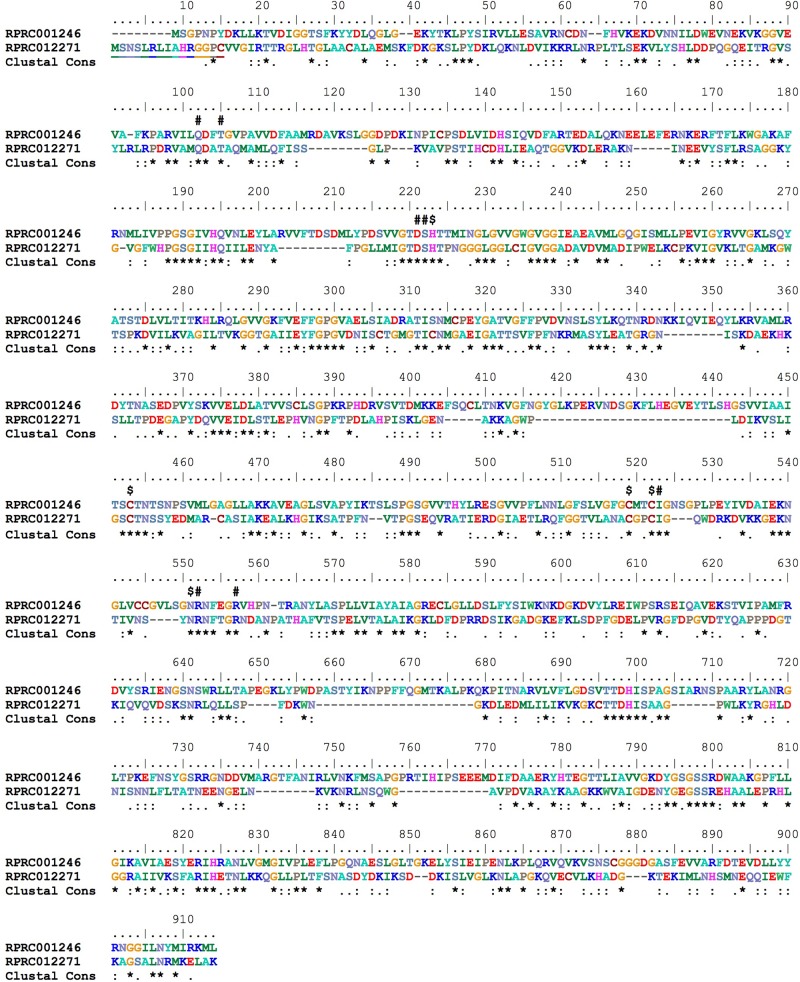
Multiple amino acid sequence alignment of *R. prolixus* cytosolic (RPRC001246) and mitochondrial (RPRC012271) IRPs. Amino acid color code generated by ClustalW within BioEdit software was used. Consensus residues (Clustal cons) were also generated by ClustalW. “#” indicates alpha-methylisocitrate binding residues and “$” Fe-S cluster binding residues. Mitochondrial targeting peptide is underlined in mitochondrial IRP (RPRC012271).

In *R. prolixus*, two genes coding for IRP-like proteins were found (RPRC001246 and RPRC012271) (**Figure 3**). RPRC012271 codes for the mitochondrial IRP and thus may not be involved in the regulation of gene expression in the cytosol. Accordingly, RpIRPs are separated into two different branches of the phylogenetic tree that includes cytosolic and mitochondrial IRPs from other insects (Supplementary Figure 3). The presence of the putative cytosolic IRP RPRC001246 (herein named IRP1), with a high degree of similarity to other insect cytosolic IRPs and of the IRE-containing mRNAs indicates that the mechanism for translational control by iron availability has been conserved in this insect.

#### Transferrin Superfamily Proteins

The transferrin family is a diverse group that comprises soluble iron-binding proteins such as serum transferrin, ovotransferrin, the antibiotic protein lactotransferrin and membrane-bound proteins such as melanotransferrins (Hentze et al., 2010; Lambert, 2012). The canonical member of this family is the vertebrate serum iron transporter transferrin. As in vertebrates, insect transferrins are monomeric proteins that bind iron with extremely high affinity and have a described role in vitelogenesis and less characterized antibiotic activity (Yoshiga et al., 1997; Harizanova et al., 2005; Dunkov and Georgieva, 2006; Ursic-Bedoya and Lowenberger, 2007).

**FIGURE 4:**
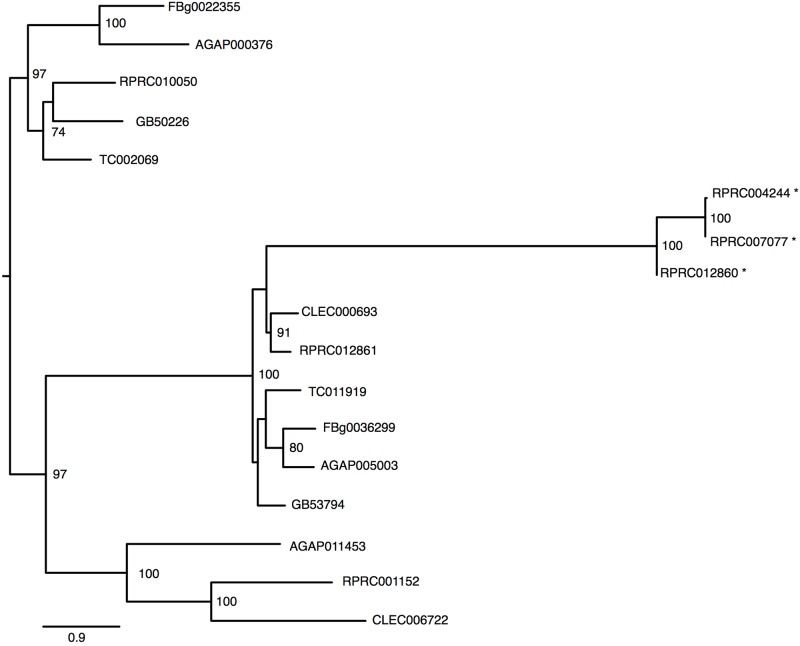
Phylogenetic analysis of Transferrin superfamily genes orthologs. Maximum-likelihood tree of transferrins orthologs. Numbers on the branches are bootstrap support values from 500 replicates. Only numbers 50% or higher are shown. “^∗^” indicate *R. prolixus* putative melanotransferrins pseudogenes. The sequence codes used were *R. prolixus* (RPRC), *D. melanogaster* (FBg), *T. castaneum* (TC), *C. lectularius* (CLEC), *A. mellifera* (GB) and *A. gambiae* (AGAP).

The *R. prolixus* genome has at least six genes that were identified as members of Transferrin superfamily (**Table 1**). The *R. prolixus* ortholog of the hemolymphatic iron-binding transferrin (Trf1) found in other insects is encoded by the RPRC10050 gene (**Figure 4**). Putative RpTrf1 presents a predicted signal peptide and the residues involved in iron binding, in the amino terminal region (N-lobe) but not in the carboxy terminal (C-lobe), suggesting that RpTrf1 is an extracellular protein capable of binding a single iron atom, as previously observed for other insects (Supplementary Figure 4). Five other genes encode for proteins that were identified as members of the transferrin superfamily. The phylogenetic tree grouped them all in the same cluster, which diverges from the cluster that comprises the insect extracellular Trf1 proteins (**Figure 4**). Two of them (RPRC012861 and RPRC001152) have a large number of introns and a predicted GPI-anchor domain, typical of the membrane-associated melanotransferrins (MelTf) (**Table 1**) (Suryo Rahmanto et al., 2012). Finally, the three other genes (RPRC012860, RPRC004244 and RPRC007077) present low similarity with the putative melanotransferrin RPRC012861 (**Figure 4**). However, none of the proteins predicted by these three presents a GPI-anchor domain (**Table 1**). Furthermore, no transcripts encoded by these genes have been found in the transcriptomes from digestive and whole-body libraries suggesting that they could be pseudogenes (Ribeiro et al., 2014).

**Table 1:** Transferrin superfamily genes identified in the *Rhodnius prolixus* genome.

Gene ID	Number of introns	Putative function	Signal peptide	GPI-anchor	Transcriptional evidence
RPRC010050	14	Hemolymphatic iron Transport (Trf1)	+	-	+
RPRC012861	10	Melanotransferin	nd	+	+
RPRC001152	12	Melanotransferin	-	+	+
RPRC012860	4	?	+	-	-
RPRC004244	1	?	-	-	-
RPRC007077	3	?	-	-	-


*“+” present, “-” absent, n/d- non-determined (truncated protein); Transcriptional evidences are according to Ribeiro et al. (2014). Signal peptide and GPI-anchor were predicted as described in the section “Materials and Methods.”*

#### Iron Transmembrane Traffic

Although the role of transferrins and ferritins in iron transport and storage has been studied in insects, very little is known concerning the mechanisms by which iron is transported across cellular membranes.

#### Malvolio/DMT-1

**FIGURE 5:**
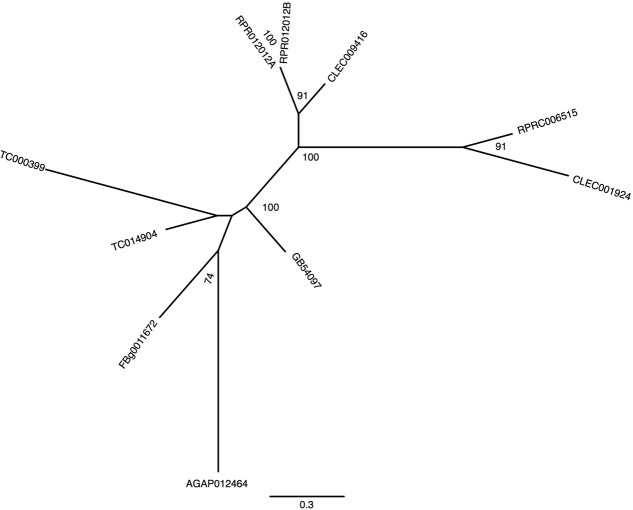
Phylogenetic analysis of malvolio orthologs. Maximum-likelihood tree of Malvolio orthologs. Numbers on the branches are bootstrap support values from 500 replicates. Only numbers 50% or higher are shown. The sequence codes used were *R. prolixus* (RPRC), *D. melanogaster* (FBg), *T. castaneum* (TC), *C. lectularius* (CLEC), *A. mellifera* (GB) and *A. gambiae* (AGAP).

In mammals, a unique protein named Divalent Metal Transporter-1 (DMT-1 or NRAMP-2) is responsible for cellular iron import from the intestinal lumen by the enterocytes (Gunshin et al., 1997). In this process, Fe^3+^ atoms provided by the diet are reduced by duodenal cytochrome b (Dcytb) to be transported to the cytosol by DMT-1 (McKie et al., 2001). Studies on the physiological role of malvolio and its involvement in iron absorption have already been performed in *D. melanogaster* (Rodrigues et al., 1995; Folwell et al., 2006) and in the new world malaria mosquito *Anopheles albimanus* (Martinez-Barnetche et al., 2007). Unlike *Drosophila* which has a single DMT-1 homolog named malvolio (MLV), *R. prolixus* presents 2 paralogous genes (RPRC006515, named MLV1 and RPRC012012 named MLV2). In addition to *R.prolixus*, the Coleopteran *T. castaneum* and the Hemipteran *C. lectularius* also have paralogs of MLV. However, *T. castaneum* MLVs are not grouped with the close–related hemiptera MLVs suggesting that a gene duplication event occurred independently among these insects (**Figure 5**). Furthermore, putative RpMLV2 has two predicted transcripts (MLV2_RA and MLV2_RB). The proteins encoded by both MLV paralogous genes have conserved transmembrane domains as shown in Supplementary Figure 5.

#### Zinc/Iron-Regulated Transporter Proteins

**FIGURE 6:**
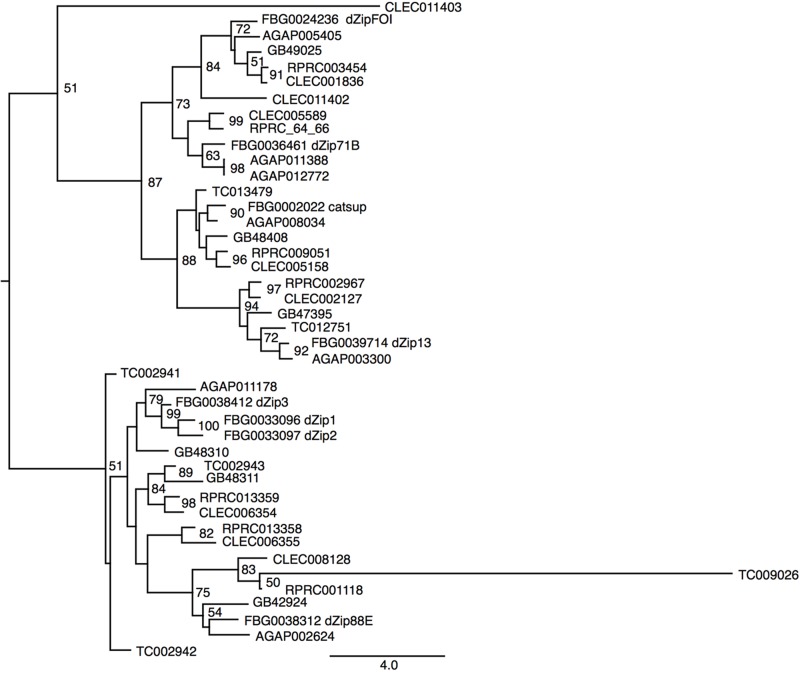
Phylogenetic analysis of ZIP orthologs. Maximum-likelihood tree of ZIP orthologs. Numbers on the branches are bootstrap support values from 500 replicates. Only numbers 50% or higher are shown. The sequence codes used were *R. prolixus* (RPRC), *D. melanogaster* (FBg), *T. castaneum* (TC), *C. lectularius* (CLEC), *A. mellifera* (GB), and *A. gambiae* (AGAP). *D. melanogaster* gene names were included in the figure to allow the identification of ZIP subfamilies.

Another class of proteins implicated in iron transport in animals is the metal transporter ZIP family. ZIP proteins are transmembrane proteins that can be localized in different compartments of the cell. In vertebrates, it has been already demonstrated that members of the ZIP family are capable of transporting both zinc and iron in different cell types (Liuzzi and Cousins, 2004; Calap-Quintana et al., 2017). So far, no description of ZIP transporters has been performed for *R. prolixus*. Seven encoding members of the ZIP family were found in the *R. prolixus* genome that are divided by homology into two major groups (**Figure 6**). RPRC003454 (ortholog of dmFOI and hZIP6/10), RPRC005566 and its paralog RPRC005564 (orthologs of dmZIP71B and hZIP5), the *D. melanogaster* catsup close-related RPRC009051 and RPRC002967 (ortholog of dmZIP99C and hZIP13) are clustered in the same group. On the other hand RPRC00118, RPRC013358 and its paralog RPRC013359 are in the second group that includes the fruit fly ZIP1, ZIP2 and ZIP3. RPRC013358 and RPRC013359, are localized side by side, in the same direction, as a cluster in the genome, suggesting that they are products of gene duplication (data not shown).

#### Mitoferrin

**FIGURE 7:**
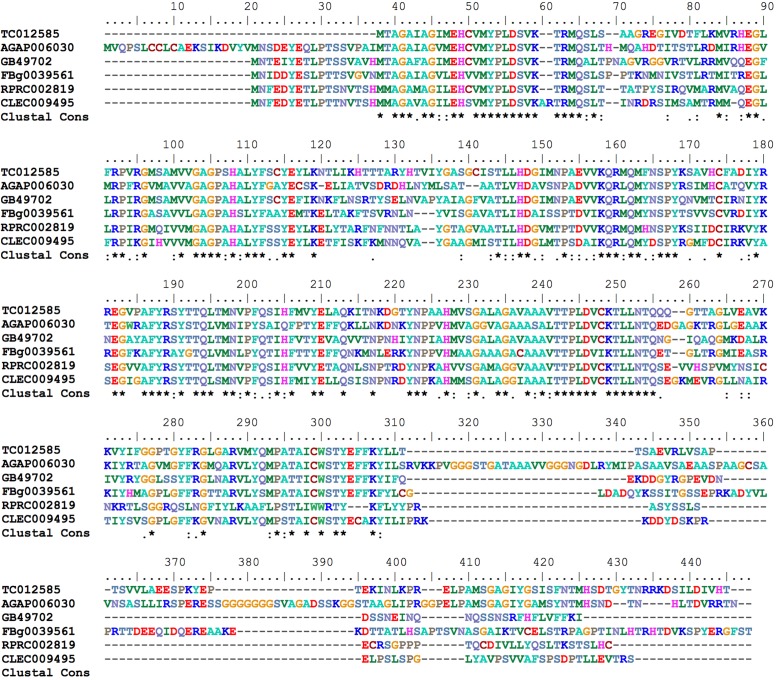
Multiple amino acid sequence alignment of *R. prolixus* mitoferrin predicted amino acid sequences with insect orthologs. Amino acid color code generated by ClustalW within BioEdit software was used. Consensus residues (“Clustal cons” line) were generated by ClustalW.

Mitoferrin (Mrfn) is a member of the mitochondrial solute carrier family. It is responsible for supplying the mitochondria matrix with iron required for heme synthesis as well as for the assembly of the iron-sulfur clusters that are components of a variety of proteins involved in energy metabolism pathways (Shaw et al., 2006; Lane et al., 2015). In vertebrates, two paralogous genes are found: mitoferrin 1 that is mainly expressed in erythropoietic cells and *mitoferrin 2* that is more ubiquitously expressed (Shaw et al., 2006). As observed in the other insect genomes that have been analyzed, the *R. prolixus* genome encodes a single putative mitoferrin (RPRC002819) with high a degree of similarity to other insect mitoferrins (**Figure 7**).

### Heme Metabolism Genes

#### Heme Biosynthesis Pathway

Most of the living organisms synthesize heme with the exception of nematodes, such as *Caenorhabditis elegans* (Rao et al., 2005) and the ticks (Braz et al., 1999; Perner et al., 2016). Genes coding for all of the enzymes of the heme biosynthesis pathway were found in *R. prolixus* genome (**Table 2**), supporting previous report showing that this insect obtains heme not only from the diet but also by synthesis *de novo* (Braz et al., 2001).

**Table 2:** *Rhodnius prolixus* genes encoding heme synthesis pathway enzymes.

Gene name	Abbreviation	Gene ID	Transcriptional evidences
5-aminolevulinate synthase	ALAS	RPRC011281	+
Delta-aminolevulinic acid dehydratase	ALAD	RPRC011504	+
Porphobilinogen deaminase	PBGD	RPRC003782	+
Uroporphyrinogen-III synthase	UROS	RPRC004613	+
Uroporphyrinogen decarboxylase	UROD	RPRC013534	+
Coproporphyrinogen III oxidase	CPOX	RPRC009673	+
Protoporphyrinogen oxidase	PPO	RPRC005054	+
Ferrochelatase	FECH	RPRC007112	+


*Transcriptional evidences are according to Ribeiro et al. (2014).*

#### Heme Degradation

**FIGURE 8:**
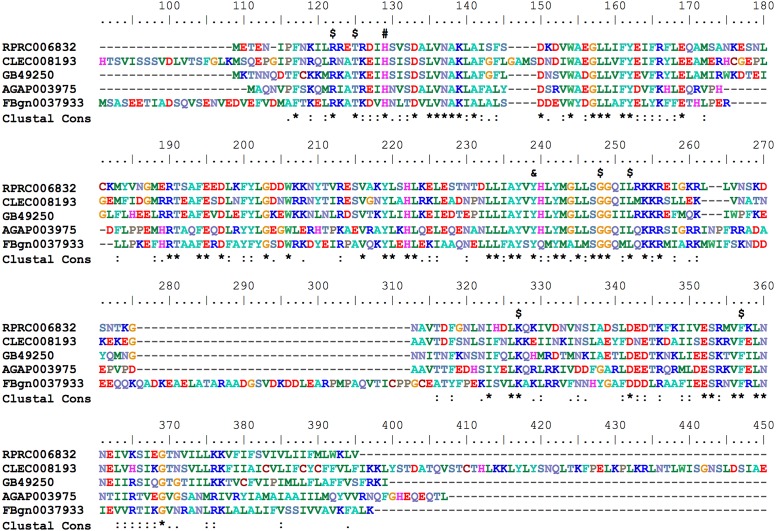
Multiple amino acid sequence alignment of *R. prolixus* Heme Oxygenase. Amino acid color code generated by ClustalW within BioEdit software was used. Consensus residues (“Clustal cons” line) were generated by ClustalW. Conserved amino acids are indicated as follows: “$” heme binding pocket, “#” histidine that coordinates to heme iron and “&” tyrosine that binds to heme propionate. The CLEC008193 sequence was much longer than the other orthologs so its unaligned N-terminal (before His91) and C-terminal (after Glu409) sequences are not shown.

Hematophagous insects have to deal with high amounts of heme released after host blood digestion in the lumen of their midguts. One of the molecular strategies involved in the cellular protection against heme-induced damage is the enzymatic degradation of heme catalyzed by HO. In most of the studied organisms, which include bacteria, plants and vertebrates, heme breakage results in formation of carbon monoxide, ferrous ion, and biliverdin IX alpha (Tenhunen et al., 1969; Wilks and Heinzl, 2014). Heme degradation pathways have been described in *R. prolixus* (Paiva-Silva et al., 2006) and in the mosquito *Aedes aegypti*, vector of Dengue, Zika and Chicungunya viruses (Pereira et al., 2007). the HO genes have not been characterized for any of these cases. A single gene encoding a HO was found in *R. prolixus* (RPRC006832) as in the other insect genome analyzed here. The predicted HO has all of the conserved residues necessary for heme interaction and degradation (**Figure 8**), as was determined in the structurally characterized recombinant *Drosophila* and human HOs (Schuller et al., 1999; Zhang et al., 2004).

#### Heme Transport_Feline Leukemia Virus Subgroup C Receptor (FLVCR)

In addition to the heme degradation catalyzed by HO in the cytosol of the *R. prolixus* gut epithelial cells, heme molecules released by hemoglobin proteolysis in the lumen of the insect gut reach the hemolymph (Dansa-Petretski et al., 1995), suggesting the existence of heme uptake and export from the gut lumen through enterocytes to the hemolymph. However, the proteins involved in this process remain unknown.

**FIGURE 9:**
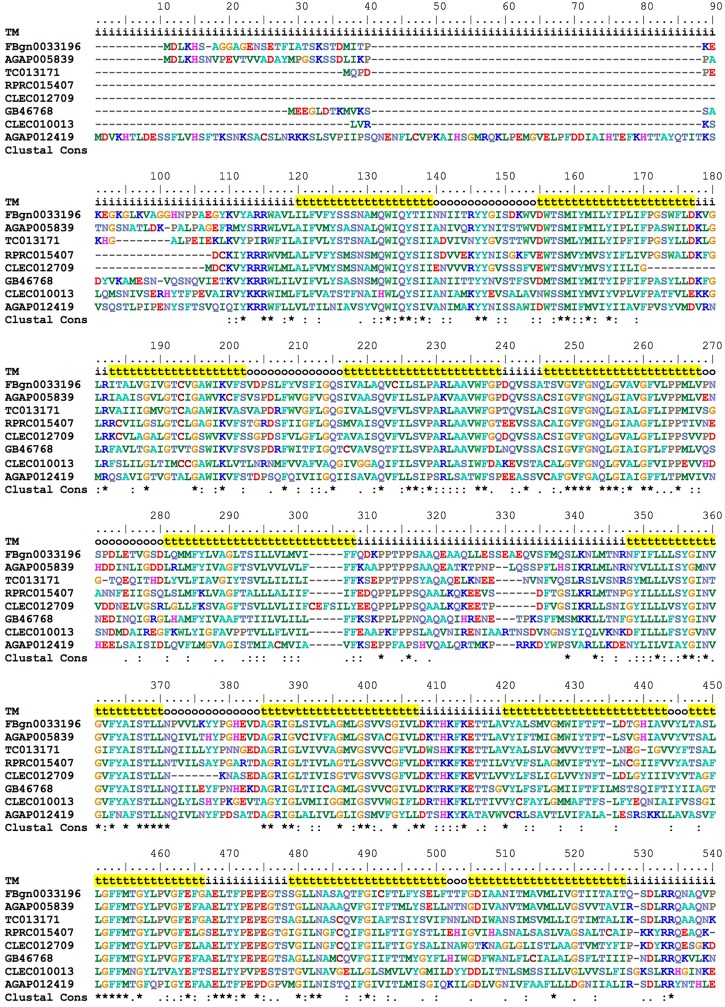
Multiple amino acid sequence alignment of *R. prolixus* FLVCR. BioEdit “ClustalW” aminoacid color code was used. Consensus residues (“Clustal cons” line) were generated by ClustalW. “TM” lines indicate transmembrane regions by yellow background “t”, inside (i) and outside (o) loops. The non-conserved C-terminal region (36 residues - position 541 to 576) was omitted to improve the conserved sequences comparison.

In vertebrates, a transmembrane protein named Feline Leukemia Virus subgroup C Receptor (FLCRV-1) has been characterized as a cell-surface heme exporter (Quigley et al., 2000, 2004). FLVCR was conserved during evolution, from animals to plants and bacteria. An FLVCR ortholog was found in the *R. prolixus* genome (RPRC015407). In the alignment performed with other putative FLVCR located in different insect genomes, we identified the 12 transmembrane canonical domains that are typical of these proteins (**Figure 9**).

#### Expression Analysis of Key Genes Involved in Iron and Heme Metabolism in the Insect Midgut Compartments

As previously mentioned, midgut epithelium is the insect tissue that primarily addresses with the high amount of blood-derived heme while avoiding heme-induced tissue damage. During the 21 days of digestion, the PM is provided with blood coming from the anterior midgut (AM), the latter being the gut segment that stores the meal after ingestion. In the PM, massive digestion of proteins and complex lipids, such as tryacilglicerol continuously occurs since the 1st day of blood ingestion (Billingsley and Downe, 1986; Terra et al., 1988; Coelho et al., 1997). During the following days, the products of digestion are absorbed by the epithelial cells localized along the PM. Given that AM and PM have distinct functions in the insect physiology, it is worth speculating that midgut cells from these two regions could differentially express iron/heme-related genes during the course of digestion. Thus, we evaluated the expression of genes involved in different aspects of iron/heme metabolism: iron storage (ferritin), heme transport (FLVCR), degradation (HO), and regulation of gene expression (IRP) in the AM and PM, during one cycle of feeding and blood digestion.

**FIGURE 10:**
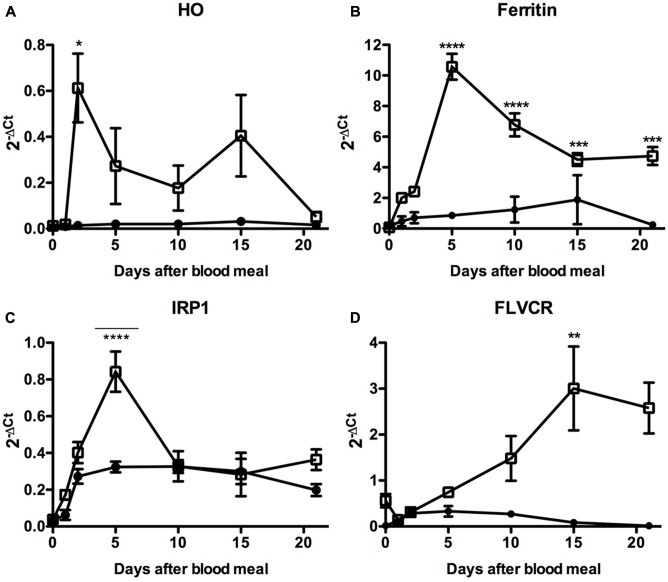
Expression analysis of key genes involved in iron and heme metabolism in the insect midgut compartments. *R. prolixus* adult females were fed on blood and the anterior and PMs were dissected immediately before (day 0) or at the indicated times after the blood-meal for qPCR analysis. Heme oxigenase **(A)**, Ferritin **(B)**, Iron Responsive Protein 1 (IRP1) **(C)**, and Feline Leukemia Virus subgroup C Receptor (FLVCR) **(D)**. The elongation factor 1 gene (RPRC007684) was used as an endogenous control (*n* = 12–15). Data shown in all graphics are mean ± SE. One-way ANOVA followed by Tukey’s multiple comparison test was used to evaluate differences in gene expression during the days after blood ingestion. ^∗^*p* < 0.05, ^∗∗^*p* < 0.01, ^∗∗∗^*p* < 0.05, ^∗∗∗∗^*p* < 0.0001. These data are resulted from three independent experiments.

In fact, we observed that the blood-meal modulated the expression of these genes, particularly in the PM where most of the blood digestion occurs and where intense release of free heme should occur (**Figure 10**). Expression of HO was induced in the PM during the very beginning of blood digestion, possibly preparing the midgut to degrade the first heme molecules that eventually reach the cytosol of the gut epithelial cells. After that, HO transcript levels were gradually reduced until day 10 when a second event of induction of HO expression was observed. Afterward the expression of this enzyme returned to the levels observed in the PM of fasting insects (day 0) (**Figure 10A**).

In agreement with this result. the expression of the secreted HCH ferritin subunit RPRC009256, the Fer subunit with the highest transcript levels in the midgut cDNA libraries (Ribeiro et al., 2014), followed HO expression, being highly upregulated in the PM (**Figure 10B**). Although less intense, a similar pattern was observed for the expression of IRP (**Figure 10C**), the protein involved in the post-transcriptional control of ferritin expression. These data suggest that both transcriptional and post-transcriptional regulation of the ferritin gene occurs in the PM, triggered by blood digestion. Interestingly, a significant modulation of IRP transcript levels was found in the AM, in the course of digestion. The heme transporter FLVCR presented a distinct gene expression profile (**Figure 10D**). The amount of FLVCR transcripts progressively increased in the PM until the 10th day, when their levels seemed to reach a plateau.

Altogether, these data reveal that intestinal cells show a complex regulation of the expression of iron/heme-related genes after a blood meal, probably to control the iron and heme availability in both the intra and extracellular celular compartment, while at the same time avoiding overload and the oxidative damage that could follow.

#### The Role of Genes Related to Iron and Heme Metabolism in Major Aspects of *R. prolixus* Physiology

**FIGURE 11:**
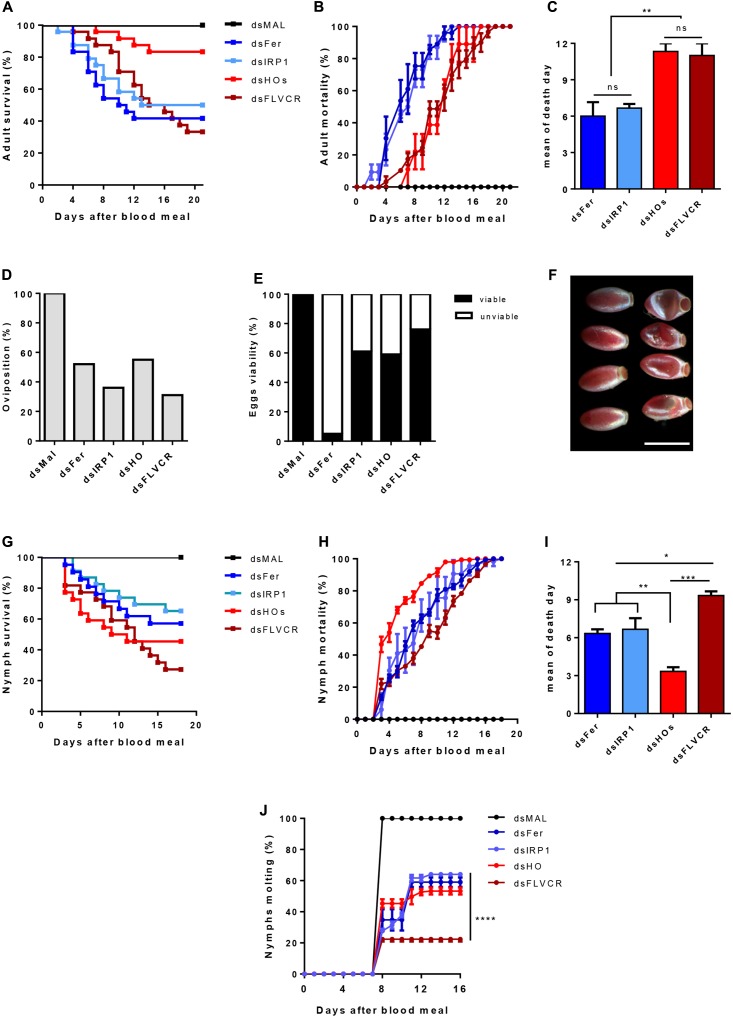
The effect of iron/heme-related genes silencing in the insect physiology. Starved adult females were injected with 1 μg of dsRNA of Ferritin (Fer), IRP (IRP1), Heme Oxygenase (HO) and FLVCR. After 48 h, the animals were fed on blood. The adults survival curves **(A)** oviposition **(D)** and eggs viability **(E)** were monitored for 21 days (*n* = 24). Nymphs of the first instar (N1) were fed on blood supplemented with dsRNAs of the same genes. The nymphs survival curves **(G)** and the molting to the N2 instar **(J)** were monitored daily for 18 days (*n* = 30). The survival curves data were replotted as % of total mortality of adults **(B)** and nymphs **(H)** to show the time course of silenced-insects mortality. The mean day of death of adults and nymphs are shown in **(C,I)**, respectively. Data shown in graphics are mean ± SE **(F)** Representative images of the phenotypic morphology of eggs laid by dsMal and dsFer-injected females (scale bar = 1,5 mm). dsMal-injected or fed insects were used as a control. Log-rank (Mantel-Cox) test was used for survival curves comparison, *p* < 0.0001 in **(A)** and *p* = 0,0001 in **(G)**. One-way analysis of variance followed by Tukey’s multiple comparison test was used to evaluate differences in the adults and nymphs mean death days, ^∗^*p* < 0.01, ^∗∗^*p* < 0.05, ^∗∗∗^*p* < 0.001. Two-way analysis of variance followed by Tukey’s multiple comparison test was used to compare molting curves of nymphs fed with iron and heme-related dsRNAs and control group (dsMAL) ^∗∗∗∗^*p* < 0.0001. All the experiments described above were independently performed at least three times.

To further determine the relevance of these genes in the physiology of the insect, we used dsRNA-mediated knockdown (KD) of the genes previously analyzed in **Figure 11**. Consistent silencing of all of the target genes was achieved, with efficiencies ranging from 60–80% and remaining for at least 7 days for both adults and nymphs (Supplementary Figure 6).

Silencing of iron-related genes (*Fers* and *IRP*) caused a significant impact on the survival of adult females (40 and 51% of survival, respectively), particularly during the 1st days of digestion when an intense heme degradation and iron release occurs (Coelho et al., 1997) (**Figures 11A,B**). In contrast, the KD of HO produced a more modest impact on survival as only 21% of the silenced insects died. In contrast, *FLVCR* KD caused intense mortality of adults (64% of mortality). However, lethality caused by silencing of the genes involved in heme metabolism was more pronounced in a later period of digestion, contrary to the early death observed after silencing of the iron-related genes (**Figures 11A–C**).

Regarding reproduction, gene silencing reduced the oviposition, at a varying rate, from 45% (dsHO) to 69% (dsFLVCR) (**Figure 11D**). Remarkably, despite the noticeable effect on oviposition, silencing of FLVCR caused only a minor impact on embryogenesis, as observed by the high rate of viable eggs produced by dsFLVCR-injected females (**Figure 11E**). On the other hand, the silencing of ferritin genes led to a reduction of 48% on oviposition but most of the laid eggs were not viable (95% of the total) (**Figures 11D,E**). The eggs laid by ferritin-silenced females had an altered morphology (**Figure 11F**), a pattern similar to eggs subjected to dehydration. This phenotype was not observed in the unviable eggs laid by females silenced for the other genes (data not shown).

To evaluate the impact of knockdown of the same genes during the insect development, nymphs (N1) were fed artificially with blood supplemented with each specific dsRNA and had their survival and molting monitored for 21 days. As observed in **Figure 11G**, the KD of all genes reduced nymphs survival. The FLVCR silencing produced the highest level of nymph mortality, as observed in adults (**Figures 11A,G**). Conversely, silencing of HO caused a more prominent effect on survival of nymphs when compared to adults (**Figures 11A,G**). Interestingly, in nymphs, HO KD led to high lethality in the early days after the blood-meal, when compared with adults (**Figures 11A–C,G–I**). Among the surviving insects, silencing of FLVCR impaired 80% of molting of N1 to N2 stage nymphs (**Figure 11J**). The KD of all of the other genes affected molting in slightly less dramatic manner, in the range of 55–65% inhibition (**Figure 11H**). Taken together, these data suggest that the control of intracellular balance of free heme is critical for the development of nymph stages.

**FIGURE 12:**
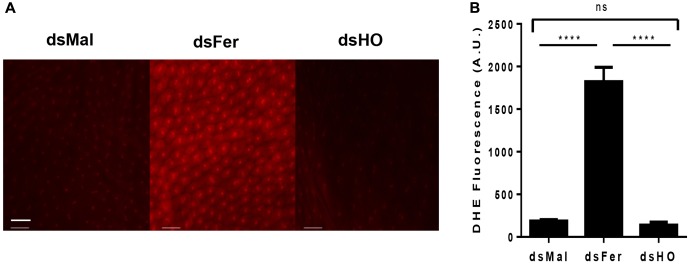
Effect of gene silencing in the production of ROS in the midgut of adult insects. Starved adult females were injected with 1 μg of dsRNA of ferritin or heme oxygenase (HO). After 48 h, the animals were fed on blood. Dissected posterior midguts were incubated at 28°C for 30 min in a solution containing 100 μM DHE. dsMal-injected females were used as a control. The tissues were analyzed by Fluorescence Microscopy (Zeiss Axio Observer Microscope) using a 20× lens with 80 ms exposure time for all conditions. **(A)** The images are representative of a total of 12 intestines analyzed. **(B)** Quantification of fluorescence using Zeiss Axio Observer Quantification software. *P* = 0.0016. One-way ANOVA with Tukey post test. ^∗∗∗∗^*p* < 0.0001 scale bar 2 μm.

Considering the early induction of HO and ferritin expression in the PM and the differences observed in the rate and the time course of mortality induced by the silencing of these genes in adults, we decided to evaluate the effect of silencing these genes on the production of ROS in the midgut epithelium after the blood repast. Knockdown of ferritin led to a dramatic increase of ROS production, observed by the increase in fluorescence of the ROS-sensitive probe DHE. On the other hand, the reduction of HO expression had no effect on the oxidative cellular status (**Figure 12**). These data showed that ferritin is a major antioxidant against iron induced damages in the midgut of fed adults. Moreover, in contrast to what is observed in mammalian models, insect HO does not contribute to the control of the redox balance in the presence of high levels of heme in fed adults.

Together these results show that imbalances in the levels of proteins involved in the control of homeostasis of heme or iron can cause extensive damage to insects in their various stages of development. These data also highlight that a fine tuning of the metabolism of these molecules is of pivotal importance to central aspects of the physiology of this blood sucking insect.

## Discussion

**FIGURE 13:**
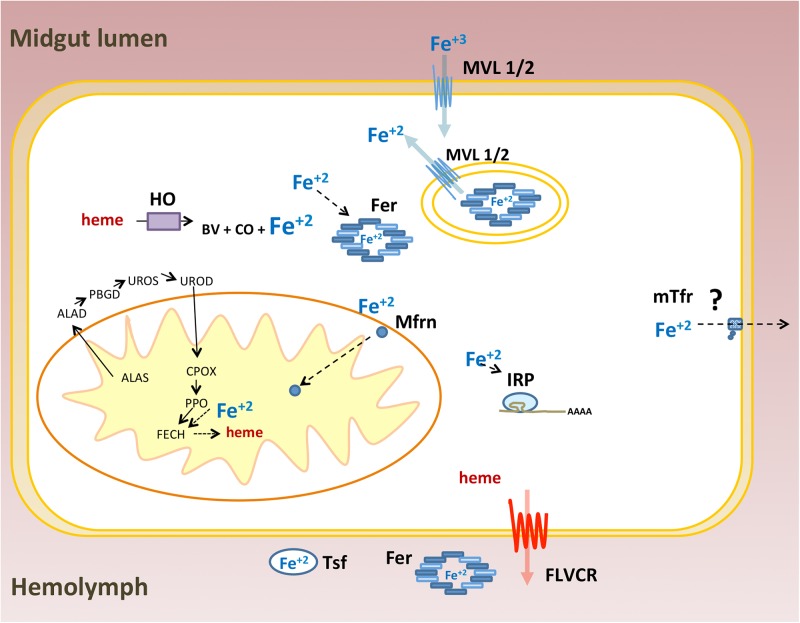
Schematic representation of iron and heme related genes identified in the *Rhodnius prolixus* genome: Ferritin (Fer), Iron Regulatory Protein (IRP), Transferrin (Tsf), Melanotransferrin (mTsf) Malvolio (MLV1/2), Mitoferrin (Mfrn), Aminolevulinic Acid Synthase (ALAS), Delta-Aminolevulinic Acid Dehydratase (ALAD), Porphobilinogen deaminase (PBGD), Uroporphyrinogen III Synthase (UROS), Uroporphyrinogen Decarboxylase (UROD), Coproporphyrinogen III Oxidase (CPOX), Protoporphyrinogen oxidase (PPOX), Ferrochelatase (FECH), Heme oxygenase (HO) and Feline Leukemia Virus C Receptor (FLVCR). ZIP proteins were ommited.

As a transition metal, iron participates in redox reactions that are essential to many physiological processes including cellular respiration, signaling and xenobiotic detoxification. Thus, iron/heme homeostasis is maintained by a vast array of proteins involved in absorption, intra and extra cellular transport, storage and efflux of these molecules (Andrews and Schmidt, 2007; Lane et al., 2015). The main iron and heme-related proteins involved in these processes were conserved in animals. Owing to the large-scale sequencing of genomes and transcriptomes, most of the proteins associated with iron and heme metabolism have been identified in many insects of different orders. However, the functional characterization and role of these proteins in general physiology have been performed mostly in the fruit fly *D. melanogaster*. In this work, 36 genes coding for proteins that control iron and heme intra and extracellular availability were revealed based on a survey of the *R. prolixus* genome. This effort was complemented by gene expression and RNAi-based experiments. A schematic representation of genes identified in this work is shown in **Figure 13**.

### Iron Metabolism Genes

Among proteins related to iron metabolism, ferritins are the most extensive studied in insects, including blood-sucking species (Pham and Winzerling, 2010). In these organisms, ferritin is implicated in the storage and the transport of dietary iron to peripheral tissues (Zhou et al., 2007). Therefore, midgut cells express and secrete ferritins loaded with iron atoms into the extracellular compartment, i.e., the hemolymph. Interestingly, the *R. prolixus* genome encodes three distinct transcripts of each secreted ferritin subunits (HCH and LCH), while all other insects already studied encode only one transcript of each subunit (**Figure 1** and Supplementary Figure 1) (Dunkov and Georgieva, 2006; Mandilaras et al., 2013; Tang and Zhou, 2013b). However, this hemipteran has similar characteristic genome organization conserved in insects: in a cluster containing a pair of HCH and LCH genes, in a tail-to-tail position (**Figure 2**) (Dunkov and Georgieva, 2006). These data lead us to speculate that those genes were originated through gene expansion of an ancient single cluster during the evolution of the blood-feeding habit in the triatomine, either as a way to increase gene expression or to allow for differential expression of each isoform, thus performing non-identical roles in the physiology of the insect. Furthermore, the organization of Fer subunits pairs in clusters, preserved during the evolution, allows the coordinated expression of both subunits, ensuring the HCH:LCH (1:1) stoichiometry observed in the ferritin multicomplexes.

In addition to the secreted HCH ferritins, this insect expresses a cytosolic HCH, as observed in vertebrates but not often in insects (**Figure 1** and Supplementary Figure 1). An exception is the *Philaenus spumarius*, a phloem-feeding Hemipteran that has a ferritin-like protein in the cytosol and nucleus of midgut cells (Collin et al., 1988). In our phylogenetic analysis, we found an ortholog of *R. prolixus* cytosolic *fer* gene in the hematophagous hemipteran *C. lectularius* (CLEC002521), suggesting that these genes may be present more frequently in insects than expected. As in vertebrates, insect cytosolic ferritins should act as intracellular chelators of free iron atoms, storing them in a non-oxidant condition.

The *Drosophila* genome contains a mitochondrial ferritin gene (Fer3HCH) (Missirlis et al., 2006). A mitochondrial HCH ferritin gene was identified in the *R. prolixus* genome. As for DmFer3HCH, the *Rhodnius* mitochondrial ferritin gene codes for a protein that has the mitochondrial targeting peptide and the conserved amino acid residues involved in ferroxidase activity and iron binding, indicating that this protein is functional. Phylogenetic analysis of ferritins genes revealed that the *R. prolixus* mitochondrial and the cytosolic ferritins are closely related, suggesting that they have a common ancestral. *Drosophila* mitochondrial ferritin has very low transcription levels in carcasses, being almost exclusive expressed in testes (Missirlis et al., 2006). Accordingly, analysis of *Rhodnius* transcriptomic data revealed that the triatomine mitochondrial ferritin transcripts were detected in whole-body but not in midgut libraries (Ribeiro et al., 2014).

The majority of the cytosolic labile pool iron is destined to mitochondria It is involved in the maintenance of intracellular energy and redox status by means of ATP and ROS production during respiration, both pathways involving the participation of iron/heme-containing proteins. Thus, proper acquisition of cytosolic iron is required to regulate mitochondrial homeostasis. Iron is a component of several Fe-S protein clusters that are part of the electron transfer chain (ETC). Furthermore, iron is a substrate of ferrochelatase that catalyzes the last step of the heme biosynthesis pathway that occurs partially in the mitochondrial matrix (Papanikolaou and Pantopoulos, 2005). The transport of iron from the cytosol to the mitochondria is performed by mitoferrin. Mitoferrin was found in the *R. prolixus* genome but in contrast to mitochondrial ferritin, mitoferrins are expressed in the midgut of *R. prolixus* (Ribeiro et al., 2014). In *D. melanogaster*, the impairment of iron transport to mitochondria by mitoferrin knockdown reduces the mortality of flies submitted to an oxidative challenge (Edenharter et al., 2017). It has recently been demonstrated that *R. prolixus* reduces mitochondrial ROS production shortly after the blood meal as a protection against oxidative damage produced by iron and heme in the midgut (Gandara et al., 2016). This unique mechanism involves the TOR pathway but it is not fully understood. It would be interesting to investigate the involvement of mitoferrin in the modulation of is mitochondrial function under this condition.

One of the major post-transcriptional regulatory mechanisms involves the Iron Regulatory protein 1(IRP)/Iron Responsive Element (IRE) system. Under intracellular iron deprivation, IRP binds to IRE stem loops localized in the 5′UTR of the target genes blocking translation. The increase of the intracellular iron levels reverts the repressor effect exerted by the IRP. In vertebrates, genes directly related to iron/heme metabolism such as ferritins, ferroportin and ALAS are under IRP control (Muckenthaler et al., 2008).

Iron responsive protein orthologs of vertebrate IRP1 have been described in insects (Zhang et al., 2001; Lind et al., 2006) that comprise hematophagous species such as *A. aegypti* and *Anopheles gambiae* (Zhang et al., 2002). *R. prolixus* has two paralogs of IRP genes that are phylogenetically included in two clades. One of them codes for a clade of mitochondrial proteins that is close related with *D. melanogaster* aconitase and must not be directly involved in iron homeostasis. The cytosolic one resembles the IRPs of other insects. IRP/IRE regulatory system seems to be operative in the triatomine, as suggested by the presence of IREs in 5′UTRs of secreted ferritin HCH and by the exceptional similarity between the expression of IRP and ferritin HCH (RPRC009256) in the midgut throughout the digestion and phenotypes produced by KD of both genes. The LCH subunits as well as the cytosolic and mitochondrial HCHs lost their IREs in the 5′UTR region of their respective mRNAs, suggesting that they are not under IRP regulation.

*Rhodnius* genome analysis identified genes belonging to the transferrin superfamily. One of these genes is an ortholog of the canonical Tsf1 genes of other insects. This gene has a many introns and codes for a secreted protein able to bind a single iron atom as do most of the insect tranferrins. Other members of the transferrin superfamily are the melanotransferrins, cell surface GPI-anchored glycoproteins (Alemany et al., 1993). High expressions of these proteins are observed in human melanomas (Suryo Rahmanto et al., 2012). Although orthologs of these proteins have already been found in many animals, including insects, their function in iron metabolism is poorly understood. An exception is the description of the role of *D. melanogaster* melanotranferrin, named Tsf2 in the assembly of epithelial septate junctions (Tiklova et al., 2010). *R. prolixus* contains two typical melanotransferrin genes, which display a GPI anchor domain, and a membrane-addressing signal peptide. Such genes are expressed in the midgut of this insect (Ribeiro et al., 2014). A second group of genes located in the same branch of the transferrin family phylogenetic tree is comprised of three genetically related genes that contain a small number of introns in their gene structure and have no GPI anchor. In addition, there are no transcriptional expression evidences. for these genes, suggesting that they may be pseudogenes. The midgut epithelium is one of the most proliferative tissue in insects. Regeneration of the epithelial monolayer, by means of cell division and replacement, require the rearrangement of the protein complexes that maintain cell-cell contact and thus the tissue integrity. Hematophagous vectors, such as *R. prolixus*, suffer a constant biotic challenge by the interaction of their intestinal epithelium with the native microbiota or with the possible pathogens acquired during the blood-meal, thus demanding constant regeneration of damaged cells. In this way the study of the role of melanotransferrins in the maintenance of intestinal epithelial integrity and in the vectorial competence of these insects seems to be of great relevance.

Iron absorption across the apical membrane of mammalian enterocytes requires a divalent iron transporter (DMT1 or NRAMP2) and a ferric reductase (Dcytb). Ferric iron atoms from the diet must be reduced by the duodenal cytochrome b (Dcytb) to be transported by DMT-1. In other cell types, such as the erythrocytes, DMT1 is also involved in the transport of iron delivered by serum transferrin. In this case, iron is reduced by the endosomal-membrane reductase STEAP3 and transported to the cytosol by DMT1 as reviewed by Muckenthaler et al. (2017). Studies on the physiological role of insect DMT-1 orthologs of Malvolio have shown that MLV is involved in iron absorption and has a role in systemic iron homeostasis (Bettedi et al., 2011; Tang and Zhou, 2013b). Mlv is highly expressed in the anterior and posterior regions of the fly midgut and mutation of this gene caused depletion of iron stores in the intestine. As in vertebrates, *R. prolixus* has two MLV paralogs (MLV 1 and MLV2), as do *C. lectularius* and *T. castaneum*. However *T. castaneum* MLVs are not grouped to these closely related hemipteran MLVs suggesting that a gene duplication event occurred independently among these insects.

Another class of proteins involved in the transport of iron in animals is the ZIP-type family of metal carriers. They are transmembrane proteins capable of transporting zinc or iron and are located in different compartments of the cell. In invertebrates, the role of these proteins is still little explored. To date, the role of ZIPs in iron transport has been demonstrated only in *D. melanogaster*. In this insect, the ZIP13 protein acts as an exporter of iron for secretory pathways (Xiao et al., 2014). Seven ZIP orthologs allocated to two different phylogenetic clades were identified in *R. prolixus*. The specificity of these proteins to transport zinc or iron and their role in the homeostatic balance of these metals still needs to be investigated in this triatomine as well as in other insects.

### Heme Metabolism Genes

Despite the high levels of heme provided by the digestion of host blood, *R. prolixus* synthesize heme in diverse tissues, including the midgut. The chemical inhibition of ALA dehydrogenase, a component of this pathway, abolishes oviposition, demonstrating the involvement of this pathway in the reproduction of this insect (Braz et al., 1999). As expected, genes encoding for all of the enzymes of the heme biosynthesis pathway were identified in the genome of *R. prolixus.*

Enzymatic degradation of heme, catalyzed by HO, is a central component in the control of heme homeostasis and is considered to be a major antioxidant mechanism against heme-induced damages, in several models. The breaking of the porphyrin ring produces the alpha isomer of biliverdin, CO and Fe^2+^. A structural and functional characterization of HO was performed in *D. melanogaster* (Zhang et al., 2004). The HO knockdown increases the total heme content in the silenced flies and causes mortality in the larvae and pupae stages (Cui et al., 2008). *R. prolixus* as well as the mosquito *A. aegypti* degrade dietary heme in the midgut epithelial cells (Paiva-Silva et al., 2006; Pereira et al., 2007; Caiaffa et al., 2010). In contrast to what has been demonstrated in other organisms, the routes of heme degradation in these hematophagous insects studied so far are very unique. In the mosquito, alpha BV produced by HO is converted into biglutaminyl-BV, possibly to increase the solubility of this molecule (Pereira et al., 2007). In *R. prolixus*, the addition of two gluthathione molecules to the heme molecule is required prior to the porphyrin ring breakage, producing a bicysteinil-gamma biliverdin (Paiva-Silva et al., 2006). Based on the structural studies of HO from other organisms (Wilks and Heinzl, 2014), the specificity of the produced isomer is given by the orientation of the porphyrin ring of the heme within the active site of the enzyme. Thus, the production of a gamma isomer of BV suggests that the HO of this triatomine exhibits structural differences in the regions that coordinate the heme-enzyme interactions. Nevertheless, the *Rhodnius* enzyme conserves the residues involved in the heme interactions, suggesting that amino acid residues interactions not predicted in other HOs may allow an unusual active site conformation that accommodates the modified heme and produces the gamma isomer of BV.

Regarding heme transmembrane transport, a gene coding an ortholog of FLVCR, a member of the major facilitator superfamily of transporters, implicated in heme export in vertebrates (Khan and Quigley, 2013) was identified in the *R. prolixus* genome. FLVCR expression in the PM of fed females, increases during the digestion process. Yang et al. (2010) demonstrated that the efflux of heme performed by FLVCR is facilitated by the interaction with hemopexin, the major heme transport protein in the plasma of mammalians. Although speculative, it is tempting to propose that RHBP, the hemolymphatic heme transporting protein (Dansa-Petretski et al., 1995), may play the same role of mammalian hemopexin, thus allowing the distribution of heme molecules from the midgut to peripheral tissue, including the growing ovaries during oogenesis.

Finally, other genes related to iron/heme metabolism found in other organisms, including the insect *D. melanogaster* remained to be identified in *R. prolixus* genome. As examples are the two multicopper oxidases MCO-1 and MCO-3 with putative reductase activity, that are implicated in iron absorption by *D. melanogaster* midgut cells (Bettedi et al., 2011). The search for orthologs of these genes in the genome of *R. prolixus* by bioinformatics tools revealed a vast number of genes with conserved domains of the multicopper oxidase superfamily. Thus, the correct identification of the respective orthologs requires the use of additional biochemical and molecular approaches. The same occurred in the search for orthologs of ABCB10, a protein that stabilizes mitoferrin during iron influx process and ABCB7, involved in Fe-S complexes biogenesis (Lane et al., 2015). In contrast, orthologs of the component of mammalian mitochondrial apparatus frataxin; as well as the Heme Regulatory Genes (HRG) proteins, involved in heme traffick in parasites, worms and vertebrates (Korolnek and Hamza, 2014) were not found. Possible reasons for these results are low conservation in gene sequence, gene loss, genome assembly errors or low genome coverage.

### Gene Expression and Silencing

Expression of ferritin, IRP, HO, and FLVCR genes, known to be capable of modulating the intracellular availability of iron and heme, is differently modulated in the insect gut throughout the digestion process. All of these genes had higher expression levels in the PM where the heme molecules are released after host hemoglobin proteolysis. It is well established in the literature that in many models (Mense and Zhang, 2006) intracellular changes in iron and heme levels can modulate the expression of several proteins related to heme metabolism, whether at transcriptional or post-transcriptional levels (Igarashi and Watanabe-Matsui, 2014). However, the signaling pathways involved in these processes are poorly understood in insects, being mostly focused on post-transcritional regulation performed by the IRP/IRE system.

An increase of IRP expression also occurs in the AM. It has already been shown that IRP can modulate genes not directly involved with iron/heme metabolism, such as citric acid cycle enzyme genes (Gray et al., 1996). We can speculate that IRP would be involved in the post-transcriptional control of genes required by the AM physiology, triggered by blood ingestion. HO has a peak of induction at the very beginning of blood digestion. In the late stage of digestion, a second event of induction is observed. The two peaks of HO expression may not have the same functional significance. The first peak is followed by an increase in ferritin and IRP transcripts levels, suggesting that in the first 10 days of blood digestion, intestinal HO activity may be an important source iron to be exported as ferritin to the peripheral tissues, particularly the growing ovary. The striking dependence of the embryo development on ferritin expression, revealed by the >95% inhibition of hatching upon ferritin silencing, provide additional support for this hypothesis. Previous studies revealed that maternally-provided heme is essential for the embryo development. Silencing of RHBP that transport heme to growing ovaries during oogenesis caused mitochondrial dysfunction and impaired embryogenesis during the early steps of embryo development (Walter-Nuno et al., 2013). However, heme molecules are not degraded during the embryogenic process, being recycled to be incorporated in newly synthesized of embryonic hemeproteins, Thus, heme is not a source of iron for the embryo. Altogether, these data indicate that the correct development of the embryos depends on an adequate iron delivery during oogenesis, performed by maternal ferritin, implicating this protein as the major transporter of iron for growing oocytes.

Of note, by day 15 after the blood-meal when there is the second peak of HO expression, most eggs (>80%) were already laid (Braz et al., 2001; Walter-Nuno et al., 2013), but there is still a significant amount of blood to be digested in the AM (Coelho et al., 1997). Therefore, the simultaneous breakdown of residual heme by HO in the intestinal epithelial cells and iron exportation to the ovary through ferritin would also prevent iron-induced damage to the gut.

Interestingly, silencing of either HO or FLVCR, both mechanisms to detoxify heme, induced death of adult females, but this occured only as a relatively late effect: >80% of the deaths occurring upon injection were after the 10th day post blood-meal. In contrast, a similar proportion (over 80%) of the lethality due to dsFerritin or dsIRP injection occurs before day 10 after the blood-meal. The late activation of FLVCR, together with the second, non-vitellogenic peak of HO, brings in the interesting possibility that these two proteins may work together, specially in the second half of digestion of the blood meal. In fact, the increase of FLVCR expression during the digestion and the severe phenotypical effects observed on survival of adults and nymphs and molting of FLVCR silenced insects. is in accordance with its role as an heme exporter, being a complementary mechanism to the route of degradation, aiming to control heme levels within enterocytes, avoiding heme overload and potential oxidative damages. In addition, the increase in heme transport from the midgut to the hemolymph can provide heme molecules to other tissues, such as the ovaries, where they are required for oocyte growth (Walter-Nuno et al., 2013).

The toxicity of iron is largely associated with its pro-oxidant properties. In the presence of oxygen, free iron promotes, through the Fenton and Haber–Weiss reactions, the generation of reactive species of oxygen, including the extremely reactive hydroxyl radical. The continuous production of ROS due to excess of iron, leads to chronic oxidative stress, causing tissue injuries and the appearance of the pathological symptoms observed with the progress of the diseases (Ryter and Tyrrell, 2000; Papanikolaou and Pantopoulos, 2005).

Not only free iron but also heme, its complex with protoporphyrin IX, is a potential pro-oxidant molecule. It promptly reacts to organic hydroperoxides producing alkoxyl or peroxyl lipid radicals, thus increasing lipid peroxidation (Ryter and Tyrrell, 2000). In addition, heme may interfere with permeability and selectivity of membranes upon insertion into the phospholipid bilayer (Schmitt et al., 1993).

This outcome points to a critical role of iron/heme-dependent ROS generation as a major potential source of oxidative damage, a conclusion that is strongly supported by the actual evaluation of oxidative species by use of the ROS-sensitive probe DHE, that revealed a dramatic increase in ROS upon ferritin silencing; this increase may be implicated as one cause of the high mortality observed in Fer and IRP silenced-insects. The apparent lack of effect of HO silencing might be due either to insuficient reduction of the enzyme or possibly to the preventive action of other antioxidant mechanisms that have been shown to operate at the same time. One is the heme aggregation (Oliveira et al., 1999, 2002) that quantitatively is the most important mechanism, sequestering most of the ingested heme (>95%) as the chemical inert crystalline hemozoin. The other would be the lowering of ROS production by endogenous pathways, particularly the mitochondria (Gandara et al., 2016). Additionally, the increase in heme concentration to levels capable of promoting ROS formation may be avoided by a compensatory efflux of heme by FLVCR.

Therefore, although there are a great number of vertebrates homologs have been found in *R. prolixus*, their biological roles and relevance have not been characterized in insects. Addressing in future research the function of these genes should result in better understanding of how insects control iron and heme homeostasis. Moreover, it may reveal new pathways and mechanisms that have allowed *R. prolixus* and other blood sucking insects to adapt to hematophagy.

## Author Contributions

AW-N, MT, PO, and GP-S designed the project and experiments. AW-N, MT, and RM performed the experiments. AW-N, MT, RM, PO, and GP-S analyzed the data. AW-N, MT, RM, and GP-S wrote the paper. AW-N, MT, RM, PO, and GP-S revised the paper.

## Conflict of Interest Statement

The authors declare that the research was conducted in the absence of any commercial or financial relationships that could be construed as a potential conflict of interest.

## Footnotes

1 www.vectorbase.org

2 https://www.ebi.ac.uk/about/vertebrate-genomics/software/exonerate

3 http://www.ncbi.nlm.nih.gov

4 http://www.cbs.dtu.dk/services/TMHMM/

5 http://www.cbs.dtu.dk/services/SignalP/

6 http://www.cbs.dtu.dk/services/TargetP/

7 https://ihg.gsf.de/ihg/mitoprot.html

8 http://mendel.imp.ac.at/gpi/gpi_server.html

9 http://ccbg.imppc.org/sires/

10 http://tree.bio.ed.ac.uk/software/figtree/
