# Supplementary material for: Silencing of Iron and Heme-Related Genes Revealed a Paramount Role of Iron in the Physiology of the Hematophagous Vector Rhodnius prolixus
Source: Front Genet. 2018 Feb 2;9:19. doi: 10.3389/fgene.2018.00019 (PMC5801409; doi:10.3389/fgene.2018.00019)
Supplement: Supplementary file 1 [file Supplementary_Material.DOCX]

Supplementary Material

SILENCING OF IRON AND HEME-RELATED GENES REVEALED A PARAMOUNT ROLE OF IRON IN THE PHYSIOLOGY OF THE HEMATOPHAGOUS VECTOR *RHODNIUS PROLIXUS*

Ana Beatriz Walter-Nuno, Mabel Taracena Oliva, Rafael D. Mesquita, Pedro L. Oliveira and Gabriela O. Paiva-Silva*

*** Correspondence:** Corresponding Author: gosilva@bioqmed.ufrj.br

# Supplementary Data

*Fasta files of repredicted genes*

Mitoferrin

>RPRC002819 repredicted based on APIS|ACYPI001051

ATGAACTTTGAAGACTATGAAACCTTGCCTACTTCCAACGTTACCAGCCACATG

ATGGCAGGAGCCATGGCTGGAATATTGGAACATTGTGTAATGTACCCACTGGATTCTGTC

AAGACCAGAATGCAGAGCCTTACAACGGCCACGCCGTACAGTATACGACAGGTGATGGCG

AGAATGGTGGCTCAAGAAGGCTTGCTGAGGCCGATTAGGGGTATGCAGATCGTAGTGATG

GGTGCTGGACCAGCCCATGCTCTATACTTCTCCAGTTATGAATATCTCAAGGAGTTATAC

ACGGCAAGGTTTAATTTCAACAACACTTTGGCCTACGGCACAGCCGGAGTTGCAGCAACG

TTACTACATGATGGAGTTATGACACCTACAGATGTTGTTAAACAACGAATGCAAATGCAT

AATAGTCCTTATAAGTCAATTATTGATTGCATTCGTAAAGTATACAAATCGGAAGGCGTC

GTTGCATTTTATAGATCTTATACAACACAATTGACAATGAATGTACCATTCCAAAGTATA

CACTTTGTTGTATATGAGACTGCGCAAAATTTATCTAATCCAACCAGAGACTATAATCCA

AAAGCGCATGTGGTAAGTGGTGCAATGGCAGGTGGTGTTGCCGCAGCGGTCACTACACCA

TTAGATGTCTGTAAGACGTTGCTCAATACTCAATCAGAGGTTGTTC

ACTCTCCAGTGATGTACAATAGTATCTGTAATAAGAGGACTTTGTCAGGAGGCCGGCAATCACTAAATGG

CTTTATCTATCTAAAGGCGGCTTTTCTTCCATCTACTTTAATATGGTGGCGCACTTACAAATTCCTATAC

TATCCCAGAGCTTCTTACAGCTCACTGTCCGAGTGCAGGTCGGGTCCACCGCCAACCCAATGCGACATAG

TACTTCTATACCAATCACTAACCAAGTCAACTTCGTTACACTGC

>RPRC002819 repredicted based on APIS|ACYPI001051

MNFEDYETLPTSNVTSHMMAGAMAGILEHCVMYPLDSVKTRMQSLTTATPYSIRQVMARM

VAQEGLLRPIRGMQIVVMGAGPAHALYFSSYEYLKELYTARFNFNNTLAYGTAGVAATLL

HDGVMTPTDVVKQRMQMHNSPYKSIIDCIRKVYKSEGVVAFYRSYTTQLTMNVPFQSIHF

VVYETAQNLSNPTRDYNPKAHVVSGAMAGGVAAAVTTPLDVCKTLLNTQSEVVHSPVMYN

SICNKRTLSGGRQSLNGFIYLKAAFLPSTLIWWRTYKFLYYPRASYSSLSECRSGPPPTQ

CDIVLLYQSLTKSTSLHC

ZIPs

>RPRC003454 1st and last exons extendended to find M and stop

MSSELVTACIFCLLCATHTHCASHHVDNDTYATIQYNDEGGGRTTINNVFIPKKVMRNVR

EIDKTNSLSKDDFIKRVFDEFGDGETLSRAGFERLILSLQRPIKNNHLEAWIYASVSVVL

ISLCGVLAVAVIPVMQKAVYHGLLQFLVALAVGTLCGDALLHLLPHAMMSINLWRGFVAA

LSVAFFYFTEKCLSLITEWKKKNQRKNKMNTIVRVMREDHGTDEESRVGEKLCKHKYSSY

PYCYGEITNSTGKFLIDTFYNNFKTVIRIREENGATKAACQDVSFIMIFTKLLSFYDNMN

YKGESLERYPIFLQLYVDTNKEEVPNSQVDYTTIILREHETHHHGHAHTHGHVHSAPGSM

GNVAWMVIMGDGIHNFTDGLAIGAAFAANIGGGISTAVAVFCHELPHEIGDFAVLLKAGM

SARQAVFYNLLSSLLCFIGMALGVVLGHNGDVKMWLFSGAAGMFLYIALVDMIPELTSSH

SKEGGSLCQCLLQLFGLMSGIGIMLIIALYEEDLMTSFQHS

>RPRC003454 1st and last exons extendended to find M and stop

ATGAGCAGCGAGTTGGTAACAGCATGTATTTTCTGTTTGCTATGTGCAACGCATA

CACATTGTGCATCACACCATGTAGATAATGATACGTATGCAACAATTCAATACAATGACG

AAGGTGGTGGTCGTACAACAATAAATAATGTATTTATACCTAAAAAAGTAATGAGAAATG

TGAGAGAAATAGATAAAACCAATAGCTTATCCAAAGATGATTTCATTAAGCGAGTTTTTG

ACGAGTTCGGCGATGGCGAAACATTGTCTAGAGCAGGATTTGAACGATTAATATTATCTT

TGCAACGTCCTATTAAAAATAATCATCTTGAAGCATGGATATATGCTAGCGTTAGCGTAG

TATTGATTAGTCTATGCGGCGTACTGGCTGTAGCAGTAATTCCTGTAATGCAGAAAGCAG

TCTACCATGGCTTGTTACAATTTTTGGTAGCCTTAGCTGTTGGTACATTATGCGGAGATG

CCTTACTCCATTTGCTTCCTCATGCAATGATGTCTATAAATCTCTGGAGAGGTTTTGTGG

CTGCTCTTTCTGTAGCTTTCTTCTATTTTACTGAAAAATGTTTATCACTTATTACAGAAT

GGAAGAAAAAAAATCAGAGGAAAAATAAGATGAATACCATTGTGAGAGTTATGCGTGAAG

ATCATGGTACAGATGAAGAATCTAGAGTTGGTGAAAAACTATGTAAACATAAGTACAGCT

CTTATCCTTATTGTTATGGAGAAATAACTAACAGTACTGGTAAGTTTTTAATAGATACCT

TTTACAATAATTTTAAAACCGTAATTCGGATAAGAGAAGAGAATGGTGCAACAAAAGCAG

CATGTCAAGATGTAAGTTTTATAATGATATTTACTAAACTGCTTAGCTTTTATGATAATA

TGAATTATAAAGGGGAAAGTTTAGAAAGATACCCGATTTTTTTACAACTTTATGTTGATA

CGAACAAGGAAGAAGTTCCTAATTCTCAAGTTGATTACACAACAATAATCTTAAGGGAAC

ATGAAACACATCATCATGGTCACGCTCATACACATGGCCATGTACATTCAGCACCTGGTT

CAATGGGCAATGTAGCATGGATGGTTATCATGGGCGATGGTATTCATAATTTCACTGATG

GTTTAGCTATTGGTGCCGCATTTGCTGCAAATATCGGAGGTGGCATTTCTACAGCAGTTG

CTGTTTTTTGTCATGAATTACCACATGAAATTGGGGATTTCGCAGTGCTATTAAAGGCTG

GAATGTCAGCTAGGCAAGCAGTATTTTACAATTTATTATCCTCTTTGCTATGTTTTATCG

GTATGGCACTTGGTGTTGTTCTTGGACATAATGGTGATGTAAAAATGTGGCTCTTTTCTG

GAGCGGCTGGAATGTTCCTTTACATCGCTCTTGTTGATATGATTCCAGAACTGACAAGCA

GTCATTCTAAAGAAGGTGGTTCACTCTGTCAGTGTTTACTTCAATTATTTGGACTTATGT

CTGGCATTGGTATTATGCTAATTATTGCTCTTTATGAAGAAGACCTCATGACATCATTCC

AGCATTCG

>RPRC005564_RPRC005566 chimera predicted based on BMOR|BGIBMGA003180

MFWFLLITIWCTISVECNHPKVDSFLQNIFDKYGNKGYMSFEGLEHLMENLGLGHLRFEE

HHNVSIHRTKDGGFQEVHDSLKLHEHNHTRSRRSYDEKEGRCLSPEELLLKYGLSPDHKV

AISPTGFLHICPGIIYQLDTRVCSTEKTSIEPDMTKEEELLVWLYGVGSIVIMSACGLLG

VLLVPLLQKALFQRVLSLLAALAVGTLSGDALLHLLPHALMGSNEDMIVLRASTTFITLL

GFFSIEAIIHGRSKA

VSTNWNRKTFTK

GKKTESENTTYAVVASSSKEKEPPEKHVDSSVTAIAWLVITGDGLHNLTDGIAVGAAFRQ

DTITGLATALAILTHELPHELGDFAVLLKGGMCIKKAIFYNILSSVLSLVGVVGGLLIGS

YGEAALWVYAITAGSFLYISLATLVPEMHKYAHSVPHTLLQLFGMFIGGGIMFIIAMYEH

TLHEVLKNN

>RPRC005564_RPRC005566 chimera predicted based on BMOR|BGIBMGA003180

ATGTTTTGGTTTTTATTAATAACAATTTGGTGTACCATTTCCGTGGAATGTAATCATCCA

AAAGTTGATAGTTTTTTACAGAATATCTTCGACAAATATGGCAATAAAGGATATATGTCA

TTTGAGGGATTGGAACATTTGATGGAAAACCTTGGTCTGGGACATCTTAGGTTTGAAGAA

CATCACAATGTCTCAATTCACAGGACTAAAGATGGAGGCTTTCAAGAAGTACACGACAGC

CTCAAGCTACATGAACATAACCATACTAGAAGCAGACGCAGTTATGATGAAAAGGAAGGT

CGATGCTTAAGCCCAGAAGAGCTGCTGCTGAAGTATGGTCTATCTCCAGATCATAAGGTT

GCAATTTCCCCGACCGGCTTTCTTCACATTTGCCCAGGAATTATATACCAACTGGATACG

AGGGTATGCTCGACCGAAAAAACATCCATAGAACCGGATATGACAAAAGAGGAAGAATTA

TTAGTGTGGCTGTACGGCGTGGGCAGTATAGTTATAATGAGTGCTTGCGGTTTACTGGGA

GTACTTCTTGTGCCGCTGTTACAGAAAGCACTGTTCCAAAGAGTACTCAGCCTGCTGGCT

GCTCTTGCCGTAGGAACTTTATCAGGAGATGCTTTACTTCATCTTTTACCACATGCATTA

ATGGGTTCTAACGAAGATATGATAGTATTGAGGGCATCTACTACTTTTATAACTCTACTG

GGATTTTTCAGTATCGAAGCGATTATTCATGGGAGAAGCAAAGCG

GTAAGTACAAACTGGAATAGAAAAACATTCACTAAA

GGTAAAAAAACTGAATCAGAAAACACTACCTACGCTGTGGTCGCTTCCAGTAGTAAGGAA

AAAGAACCGCCGGAAAAACATGTGGACAGCTCTGTTACAGCGATTGCCTGGTTAGTGATA

ACAGGCGATGGACTGCACAATCTGACAGATGGAATTGCCGTGGGTGCCGCATTCCGACAA

GACACTATTACCGGCTTAGCTACAGCGTTAGCCATATTAACACATGAACTGCCGCATGAA

CTAGGTGACTTCGCAGTACTATTGAAAGGTGGTATGTGCATTAAGAAAGCTATTTTCTAC

AACATCCTGTCTTCAGTTCTCAGTCTAGTTGGTGTCGTGGGAGGTTTACTGATAGGAAGT

TATGGTGAAGCTGCACTTTGGGTGTACGCTATTACAGCTGGCTCGTTTCTTTACATTTCA

TTGGCAACTTTGGTACCAGAAATGCACAAATATGCACATAGCGTGCCACATACTCTGTTA

CAATTATTTGGCATGTTCATCGGTGGCGGAATTATGTTCATCATTGCCATGTACGAGCAT

ACCTTACATGAAGTATTGAAGAACAATTGA

>RPRC009051 predicted by CLEC|CLEC005158 and manual curation

ATGTTTCCTAAAGTGGAAAGTGGCTGCAA

TAATGCACTACTCAAAAACTGTCGGGCATTGTATTTTTTAGTTTTGCTGTGTTTCTTAAG

TTTGTTATTTTTGAATTTTCCTTTATTGTGCACTGGTGAT

GGACATGGCCATTCTCACCAACACTCAGAAGAACACCCTTCCTATAAATATTCCAGAGAA

GCAAATCTTCCGCTGAATGATCAAGATGATATTCAAAAGAAACCGGTAGGAGTTGGAAAA

ATTGATCGTGATGCTTTGTGGTTTAATGCACTGGGCTCTACACTGTTGATTAGTATAGCT

CCATTTTTTATTCTCTTTCTGGTCCCCTTAGATAACAGTTCAGAACGAGAACCATTGCTA

AAGATACTACTGAGCTTTGCAGCCGGTGGCCTTTTGGGAGATGCTTTTCTCCATCTCATT

CCTCATGCCTTACTTGGACATACTCATGAAAATCATGGAGGCACTCACCAGGAACATGAT

CATGAGGATACTCATGATCATTCACACGATATGAACGTAGGTTTGTGGGTGTTAAGCGGT

ATACTTATTTTCTTGTTTGTGGAGAAGTTTGTACGTTTAATGAAGAATGAACATGGACAT

TCACATTCATCTAAGGCACCCAGTGAAAAAAACCACAAATCAGATTCAAAGAGTAAATCT

GGGCAAAAGAGAACTGCAAAAGAATCTGGAAATGACATTAAGATTGCCGGCTATTTAAAT

CTTGCGGCTGATATAACTCACAACTTCACTGACGGCTTGGCAATTGGCGCCTCTTATTTA

GCGGGCAGGAATGTTGGTATAGTTACCACAATAACTATTCTTCTGCATGAAGTTCCTCAT

GAAATCGGAGATTTTGCAATACTCATCAAATCCGGGTGTTCTCGGAAAAAGGCAATGTAT

CTTCAACTTCTGACGGCTGTTGGTGCCCTTTCAGGAACTGTCGTATCTTTATTGGCAGAA

GGCTATGATGAGATGGCTACGGCATGGATATTACCATTAACGGCTGGAGGGTTTATATAT

ATAGCTACAGTATCAGTTCTGCCAGAATTATTGTCCGACACAAAATTGTGGCAGTCAATT

AAAGAAATATTGGCACTTCTCTTAGGTGTATATATGATGGTGCTGATTGCCTCATTTGAA

TAA

>RPRC009051 predicted by CLEC|CLEC005158 and manual curation

MFPKVESGCNNALLKNCRALYFLVLLCFLSLLFLNFPLLCTGDGHGHSHQHSEEHPSYKY

SREANLPLNDQDDIQKKPVGVGKIDRDALWFNALGSTLLISIAPFFILFLVPLDNSSERE

PLLKILLSFAAGGLLGDAFLHLIPHALLGHTHENHGGTHQEHDHEDTHDHSHDMNVGLWV

LSGILIFLFVEKFVRLMKNEHGHSHSSKAPSEKNHKSDSKSKSGQKRTAKESGNDIKIAG

YLNLAADITHNFTDGLAIGASYLAGRNVGIVTTITILLHEVPHEIGDFAILIKSGCSRKK

AMYLQLLTAVGALSGTVVSLLAEGYDEMATAWILPLTAGGFIYIATVSVLPELLSDTKLW

QSIKEILALLLGVYMMVLIASFE

>RPRC013359 repredicted extending N and C temini

MKYADFLPINDTSVDSEEQNALFMAKVSSMAVLGLLSFILGTLPIKLASCLRWKVQTIDN

QGRHPLIISLLLCFGGGVLLNTTFMHLLPEVRENMNDVIESDTLPEFITNSSLNLPELLF

CLGFFLVYMIEEIVHALMHHHDHRDDLEVLHRSLSVRKCTMIPRISLSKPSPPSTITGST

QVLIRDPSLQTDSLGQITLPTGAGSDPSDTSSAAPSETKSTVVKSFRGLLAVIALSFHAV

FEGLAIGLEQEPSSVWYLCAAVATHKLVIAFCIGVELVSSRTKTCLIALYMATFAVVSPL

GIAIGLVMSFEDALEHRNGLPLASVVLQGMAAGTLLYVVFFEVLQREKSNARHGLIQLFS

IFTGFIVLLALSFFSEYFIFFLMVIIIV

>RPRC013359 repredicted extending N and C temini

ATGAAGTATGCTGACTTTCTTCCAAT

TAACGACACTTCTGTTGACAGTGAAGAACAAAACGCCTTATTTATGGCAAAAGTGTCTTC

CATGGCGGTACTCGGGTTATTGTCATTTATTTTGGGTACATTACCAATTAAATTAGCCAG

CTGTCTACGTTGGAAAGTGCAAACAATAGATAACCAGGGACGACATCCGCTCATCATTTC

GTTGTTGTTATGTTTTGGCGGCGGAGTCCTCTTAAATACTACATTCATGCATTTATTACC

GGAAGTAAGGGAAAATATGAATGATGTAATTGAATCTGACACATTACCCGAATTTATTAC

TAATTCCAGCTTGAATTTACCAGAATTATTATTCTGTTTGGGCTTCTTCTTGGTGTATAT

GATTGAAGAGATTGTGCATGCACTGATGCATCATCATGACCACAGAGACGATCTGGAAGT

GTTGCACAGGTCGCTGTCAGTTAGAAAGTGCACCATGATACCAAGAATTTCGCTATCAAA

GCCGTCACCTCCCTCCACGATAACAGGTAGCACGCAGGTACTGATACGAGACCCTTCGCT

CCAGACAGACTCCCTAGGGCAGATCACACTACCAACAGGAGCCGGTTCTGATCCATCTGA

TACATCTTCCGCTGCGCCTTCTGAAACTAAGAGCACTGTCGTGAAATCATTTAGAGGATT

GTTAGCTGTGATAGCTCTATCATTTCACGCTGTGTTCGAAGGTCTGGCCATTGGTTTGGA

ACAGGAACCATCTAGTGTGTGGTACTTATGCGCAGCAGTCGCAACACATAAACTAGTTAT

CGCATTCTGCATCGGCGTGGAATTGGTTAGCTCTCGAACTAAAACTTGTTTGATAGCTTT

GTACATGGCAACATTTGCAGTTGTTTCGCCGCTTGGTATCGCCATCGGTCTGGTCATGTC

TTTTGAGGATGCGTTGGAGCATAGAAATGGTCTGCCTCTGGCGTCAGTAGTGCTTCAGGG

AATGGCTGCCGGAACCTTGCTCTACGTCGTCTTCTTCGAAGTTCTGCAGAGAGAGAAGTC

AAATGCACGCCACGGTCTCATACAGTTGTTCTCCATCTTCACCGGCTTCATCGTACTACT

TGCCCTAAGCTTCTTTAGTGAGTATTTTATCTTCTTTCTTATGGTTATCATCATCGTTTA

# Supplementary Figures and Tables

## Supplementary Tables

**Supplementary table 1:** Oligonucleotide sequences used for amplification of dsRNA synthesis templates. Oligonucleotides were designed using the Program Primer 3 version 0.4.0 (<http://bioinfo.ut.ee/primer3-0.4.0>). T7 promoter sequence necessary for transcription is underlined. Gene sequences and accession numbers used are as deposited in Vectorbase database (<https://www.vectorbase.org/>).

| Name | Target gene | Sequence |
| --- | --- | --- |
| T7RpFerFw | RPRC009256 | TAATACGACTCACTATAGGCCCAGATTCCAACAGAATGG |
| T7RpFerRv | RPRC009256 | TAATACGACTCACTATAGGCCCTCTGGCCTGTGTATTGT |
| T7RpIRP1Fw | RPRC001246 | TAATACGACTCACTATAGGGGGTGGCAATGCTGAGAGAT |
| T7RpIRP1Rv | RPRC001246 | TAATACGACTCACTATAGGGTGTGACATCTCCAGAGCCG |
| T7RpHOFw | RPRC006832 | TAATACGACTCACTATAGGGTGGAACAAGCTATGTCTGCAAAT |
| T7RpHORv | RPRC006832 | TAATACGACTCACTATAGGGTCTTCGTCAAGTGAATCAGCGA |
| T7RpFLVCRFw | RPRC015407 | TAATACGACTCACTATAGGGGGTTCTGTTGTTTGTGGTTT |
| T7RpFLVCRRv | RPRC015407 | TAATACGACTCACTATAGGGTTGAGCTTCCTGCCTTCTGT |

**Supplementary table 2:** Oligonucleotide sequences used for Real Time PCR assays. Oligonucleotides were designed using the Program Primer 3 version 0.4.0 (<http://bioinfo.ut.ee/primer3-0.4.0>). Gene sequences and accession numbers used are as deposited in Vectorbase database (<https://www.vectorbase.org/>).

| Name | Name | Sequence |
| --- | --- | --- |
| qRpFerFw | RPRC009256 | AAAGAATGCGAAGGTTCACG |
| qRpFerRv | RPRC009256 | GCTTTTCCAGCTAAATCCCTCTG |
| qRpIRPFw | RPRC001246 | ACGACAGTTAGGAGTGGTAGGT |
| qRpIRPRv | RPRC001246 | AGAAACCAACAGTGGCACCAT |
| qRPHOFw | RPRC006832 | CCAGAGACATTCATTCTGCATTTTAG |
| qRPHORv | RPRC006832 | GTCCTCTCCATGCCATTCAC |
| qRpFLVCRFw | RPRC015407 | CGGCTCATGACAAATCCAGG |
| qRpFLVCRRv | RPRC015407 | AATCCAATCCTCCCTGCGTC |
| qRpEF-1Fw | RPRC007684 | GATTCCACTGAACCGCCTTA |
| qRpEF-1Rev | RPRC007684 | GCCGGGTTATATCCGATTTT |

## Supplementary Figures


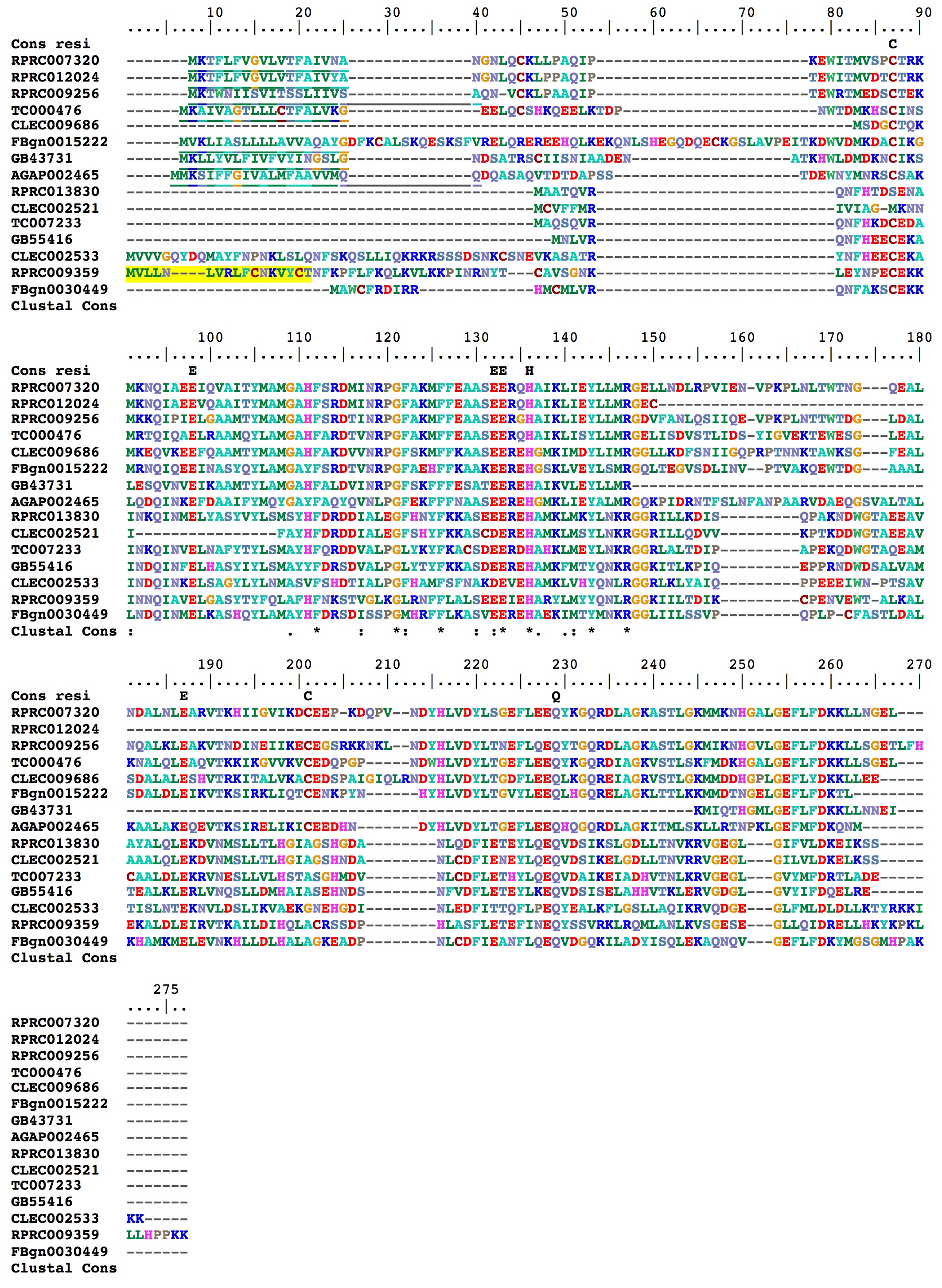


**Supplementary Figure 1A:** **Multiple amino acid sequence alignment of *R. prolixus* Ferritins HCHs with insect orthologs.** Aminoacid color code generated by ClustalW within BioEdit software was used. Consensus residues (Clustal cons) was generated by ClustalW. Amino acid residues involved in ferroxidase activity are indicated in bold (cons resid). Signal peptides for secretion are underlined and mitochondrial-target peptide is in yellow background. The sequence ID were *R. prolixus* (RPRC), *D. melanogaster* (FBgn), *T. castaneum* (TC), *C. lectularius* (CLEC), *A. mellifera* (GB) and *A. gambiae* (AGAP).


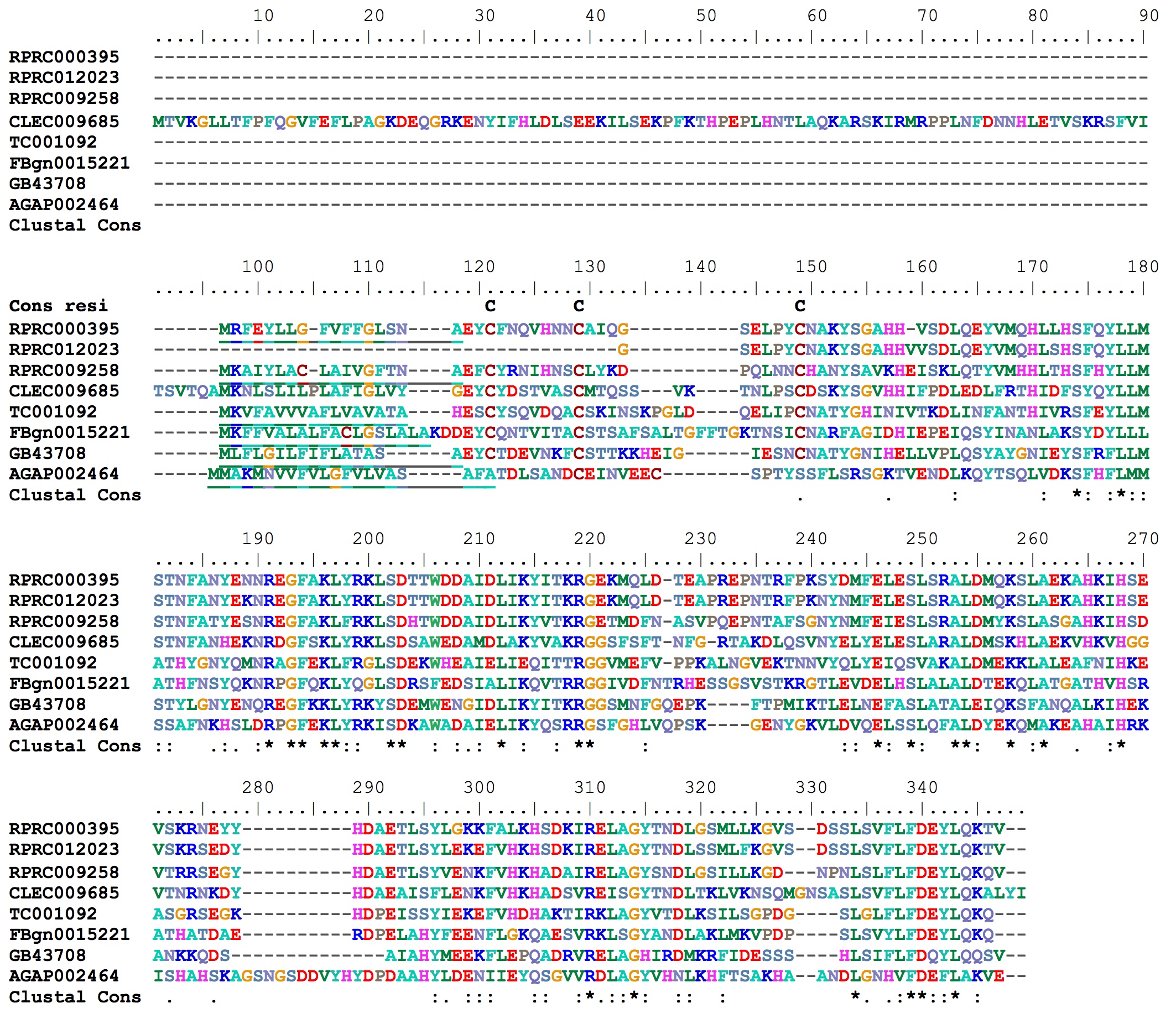


**Supplementary Figure 1B:** **Multiple amino acid sequence alignment of *R. prolixus* Ferritins LCHs with insect orthologs.** Aminoacid color code generated by ClustalW within BioEdit software was used. Consensus residues (Clustal cons) was generated by ClustalW. Conserved cysteine residues are indicated in bold (cons resid). Signal peptides for secretion are underlined. The sequence ID were *R. prolixus* (RPRC), *D. melanogaster* (FBgn), *T. castaneum* (TC), *C. lectularius* (CLEC), *A. mellifera* (GB) and *A. gambiae* (AGAP).


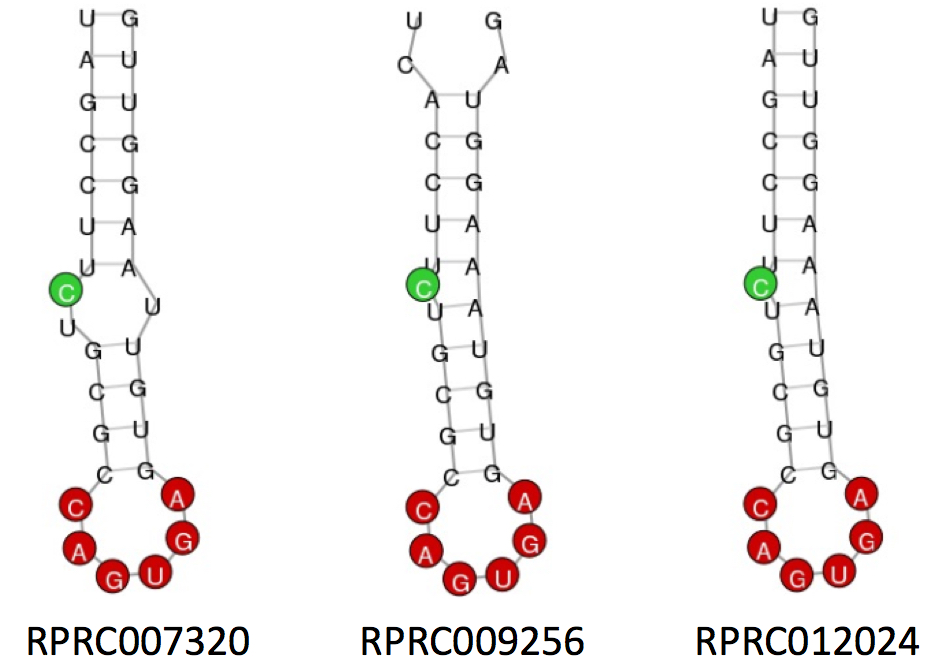


**Supplementary Figure 2: IRE Secondary structures of secreted Ferritin HCH genes.** Prediction of the secondary structure of the IREs used the 5'UTRs of the secreted Ferritin HCH genes. The residues marked in red indicate the canonical type 1 loop. The cytosine base at position 8 (in green) is involved in the interaction with the IRPs. IREs are located in 5 ́UTR of secreted Fer HCH cDNA at the following positions : -119 to -87bp in RPRC007320; -118 to -87bp in RPRC009256 and -120 to -89bp in RPRC012024 from the start codon (AUG) for translation.


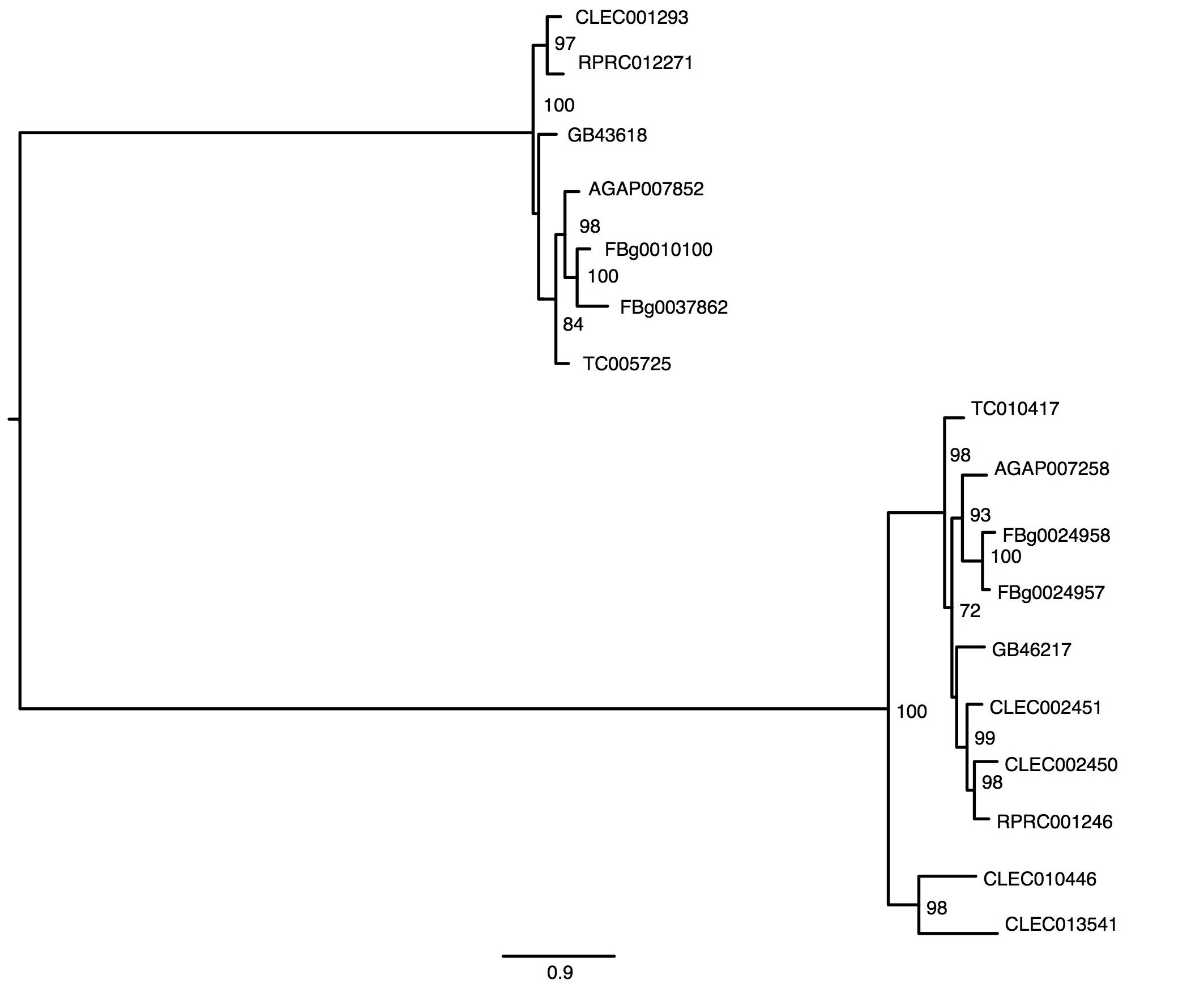


**Supplementary Figure 3**: **Phylogenetic analysis of IRPs insect orthologs.** Maximum-likelihood tree of IRP insect orthologs. Numbers on branches are bootstrap support values from 500 replicates. Only numbers 50% or higher are shown. The sequence codes used were *R. prolixus* (RPRC), *D. melanogaster* (FBg), *T. castaneum* (TC), *C. lectularius* (CLEC), *A. mellifera* (GB) and *A. gambiae* (AGAP).


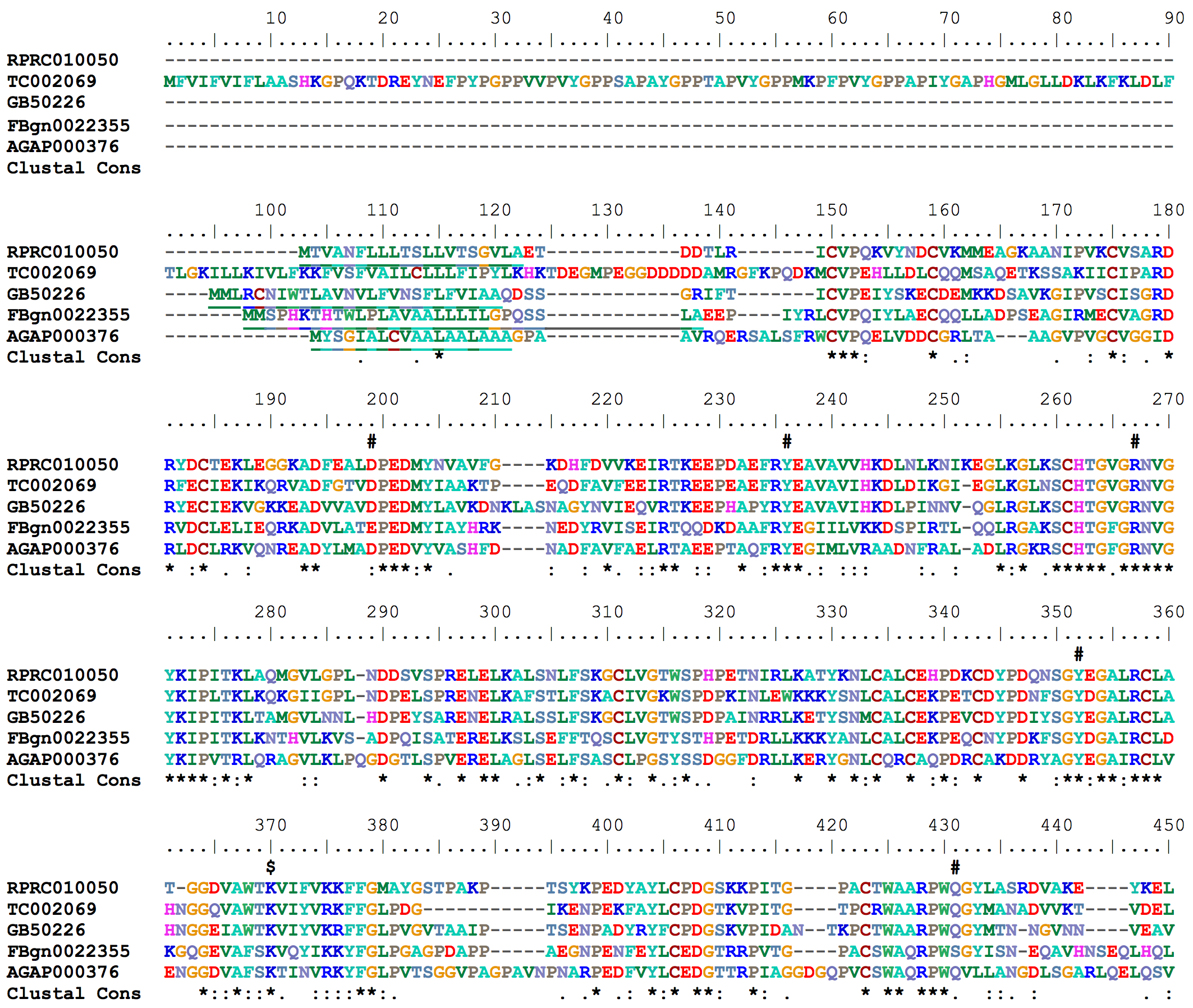


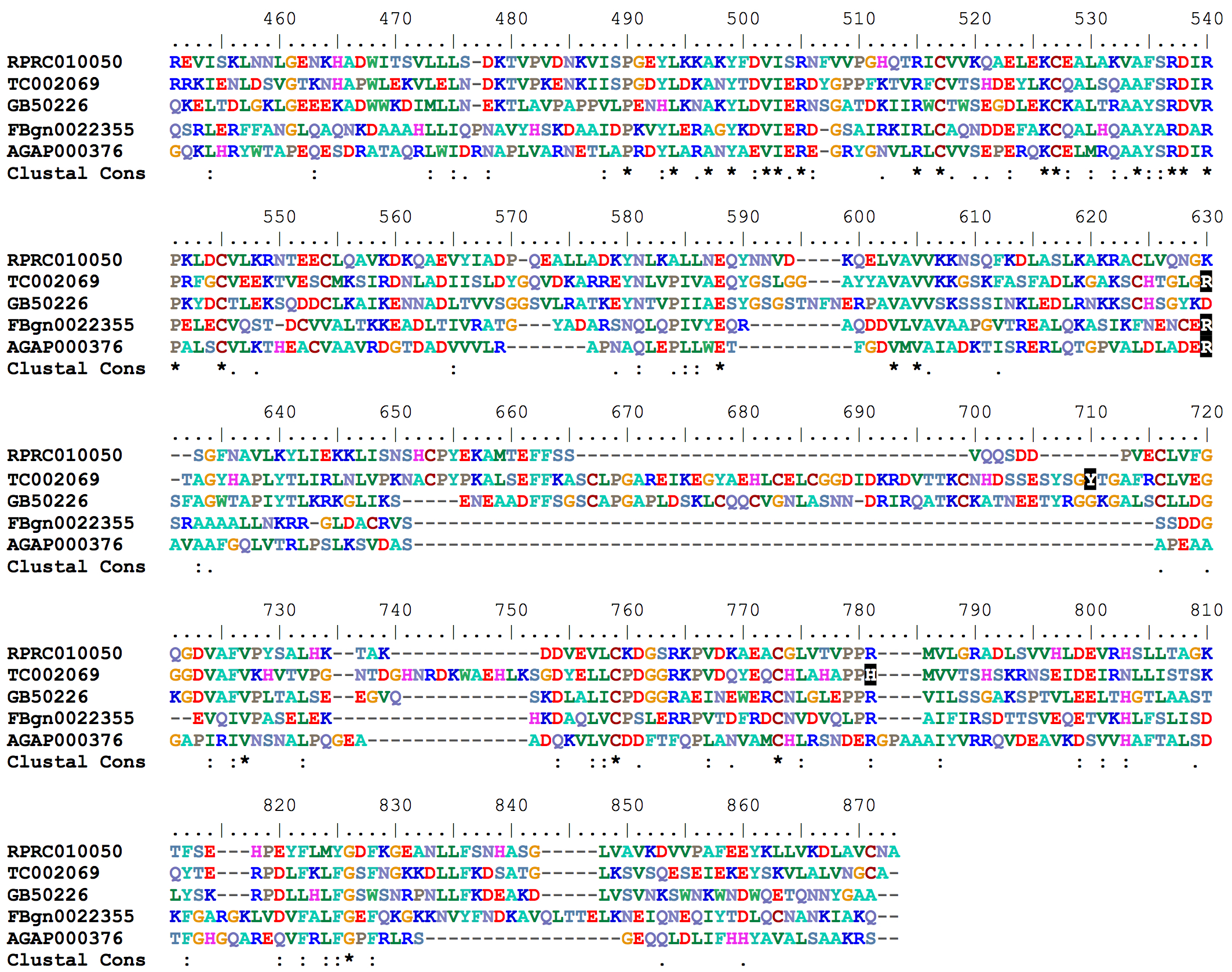


**Supplementary Figure 4:** **Multiple amino acid sequence alignment of *R. prolixus* Transferrin 1 (Tsf-1) with insect orthologs.** Aminoacid color code generated by ClustalW within BioEdit software was used. Consensus information (Clustal cons) was generated by ClustalW. Conserved amino acids are indicated as follows: “#” N-lobe residues involved in iron binding., ”$” Conserved lysine responsible by iron release. C-lobe residues are marked with black background; Signal peptides for secretion are underlined.The sequence ID were *R. prolixus* (RPRC), *D. melanogaster* (FBgn), *T. castaneum* (TC), *C. lectularius* (CLEC), *A. mellifera* (GB) and *A. gambiae* (AGAP).


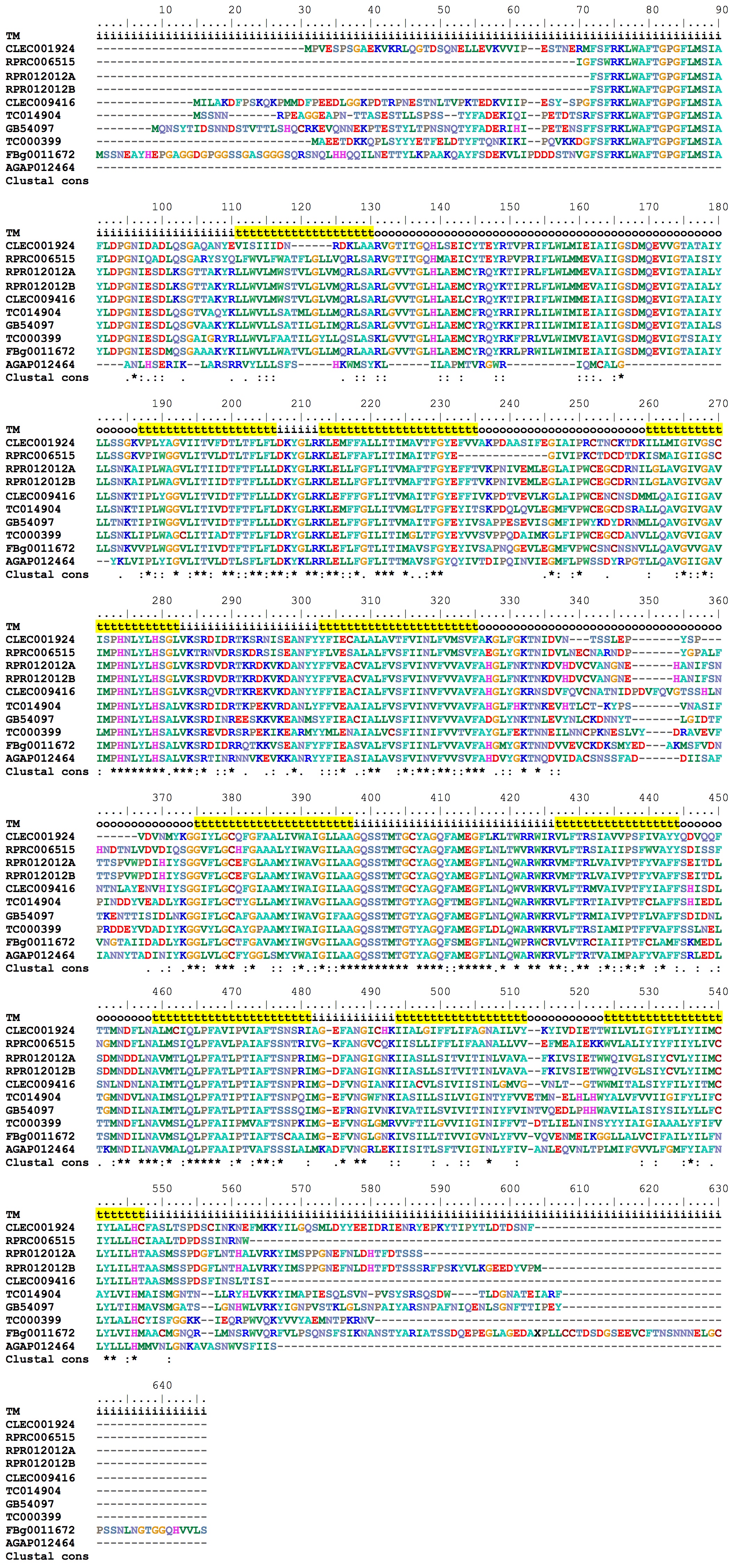


**Supplementary Figure 5: Multiple amino acid sequence alignment of *R. prolixus* Malvolio with insect orthologs**. Aminoacid color code generated by ClustalW within BioEdit software was used.. Consensus information (“Clustal cons” line) was generated by Clustalw. “TM” lines indicate transmembrane regions by yellow background “t”, inside (i) and outside (o) loops. The sequence codes used were *R. prolixus* (RPRC), *D. melanogaster* (FBgn), *T. castaneum* (TC), *C. lectularius* (CLEC), *A. mellifera* (GB) and *A. gambiae* (AGAP).

**Supplementary Figure 6:** dsRNA-mediated Knockdown of heme / iron-related genes on days post feeding. Adult females were injected with 1 μg of dsRNA for the following genes Fer (dark blue); dsIRP1(light blue), dsHO (red) and dsFLVCR (dark red). Insects were fed on blood 48 hour after dsRNA injection. The females were dissected and the total RNA was extracted from posterior midgut on the different days after feeding. The levels of expression of the genes were determined by real-time PCR. The Elongation factor 1 (EF-1) gene was used as endogenous control. The result was normalized in relation to the animals injected with dsMAL (dashed line). Data on graph is mean ±SE of three independent biological replicates (pool of 4-5 midguts), each one analyzed in triplicate. A two-way ANOVA with Bonferroni post-test was used to evaluate differences between the expression of the different genes and the control group at different times (**** P<0.0001).
