# Supplementary material for: Silencing of Iron and Heme-Related Genes Revealed a Paramount Role of Iron in the Physiology of the Hematophagous Vector Rhodnius prolixus
Source: Front Genet. 2018 Feb 2;9:19. doi: 10.3389/fgene.2018.00019 (PMC5801409; doi:10.3389/fgene.2018.00019)
Supplement: Supplementary file 2 [file Figure_S1.DOCX]

Supplementary Material

SILENCING OF IRON AND HEME-RELATED GENES REVEALED A PARAMOUNT ROLE OF IRON IN THE PHYSIOLOGY OF THE HEMATOPHAGOUS VECTOR *RHODNIUS PROLIXUS*

Ana Beatriz Walter-Nuno, Mabel Taracena Oliva, Rafael D. Mesquita, Pedro L. Oliveira and Gabriela O. Paiva-Silva*

## Supplementary Figures


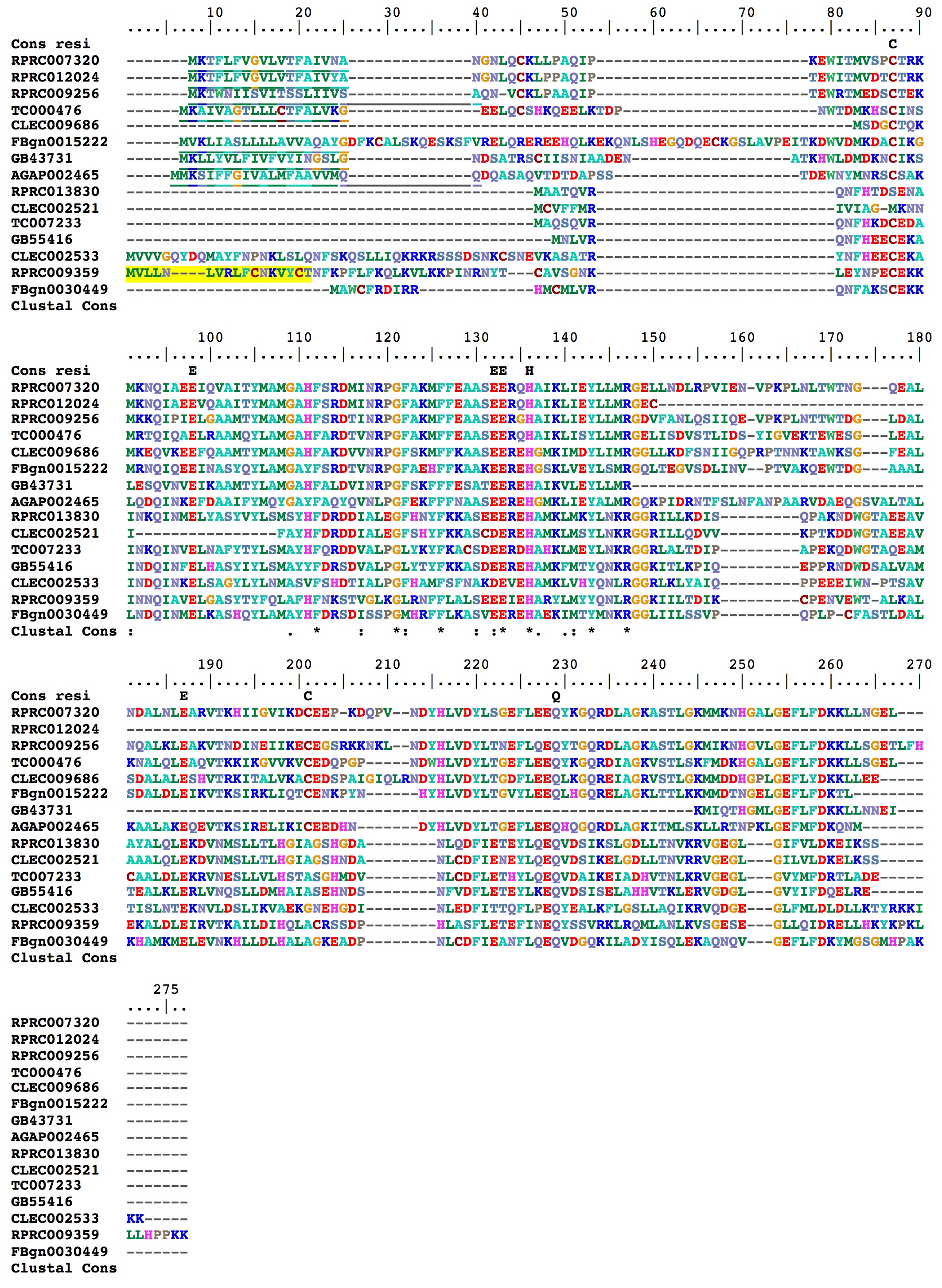


**Supplementary Figure 1A:** **Multiple amino acid sequence alignment of *R. prolixus* Ferritins HCHs with insect orthologs.** Aminoacid color code generated by ClustalW within BioEdit software was used. Consensus residues (Clustal cons) was generated by ClustalW. Amino acid residues involved in ferroxidase activity are indicated in bold (cons resid). Signal peptides for secretion are underlined and mitochondrial-target peptide is in yellow background. The sequence ID were *R. prolixus* (RPRC), *D. melanogaster* (FBgn), *T. castaneum* (TC), *C. lectularius* (CLEC), *A. mellifera* (GB) and *A. gambiae* (AGAP).


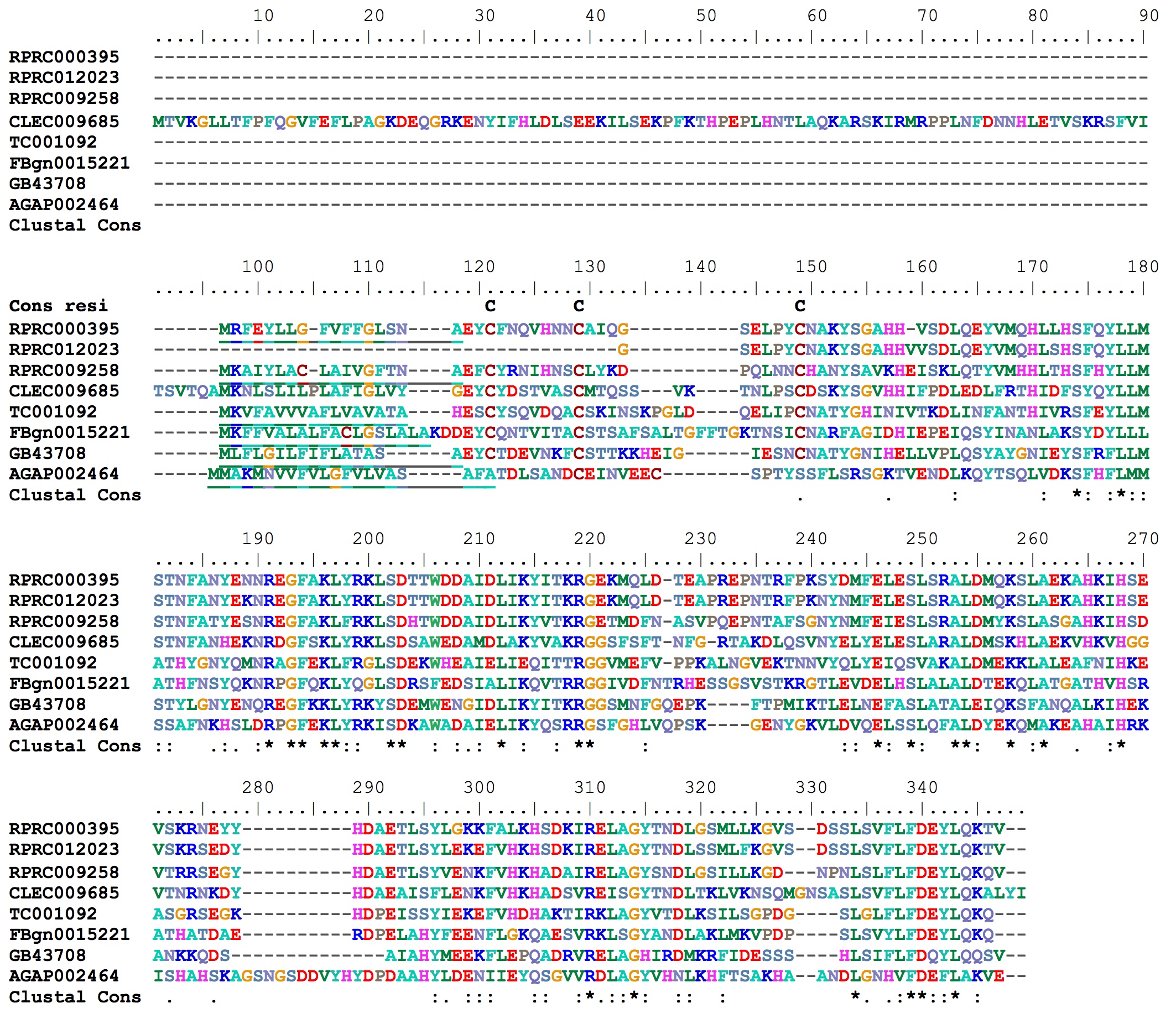


**Supplementary Figure 1B:** **Multiple amino acid sequence alignment of *R. prolixus* Ferritins LCHs with insect orthologs.** Aminoacid color code generated by ClustalW within BioEdit software was used. Consensus residues (Clustal cons) was generated by ClustalW. Conserved cysteine residues are indicated in bold (cons resid). Signal peptides for secretion are underlined. The sequence ID were *R. prolixus* (RPRC), *D. melanogaster* (FBgn), *T. castaneum* (TC), *C. lectularius* (CLEC), *A. mellifera* (GB) and *A. gambiae* (AGAP).
