# Supplementary material for: Silencing of Iron and Heme-Related Genes Revealed a Paramount Role of Iron in the Physiology of the Hematophagous Vector Rhodnius prolixus
Source: Front Genet. 2018 Feb 2;9:19. doi: 10.3389/fgene.2018.00019 (PMC5801409; doi:10.3389/fgene.2018.00019)
Supplement: Supplementary file 3 [file Figure_S2.DOCX]

Supplementary Material

SILENCING OF IRON AND HEME-RELATED GENES REVEALED A PARAMOUNT ROLE OF IRON IN THE PHYSIOLOGY OF THE HEMATOPHAGOUS VECTOR *RHODNIUS PROLIXUS*

Ana Beatriz Walter-Nuno, Mabel Taracena Oliva, Rafael D. Mesquita, Pedro L. Oliveira and Gabriela O. Paiva-Silva*


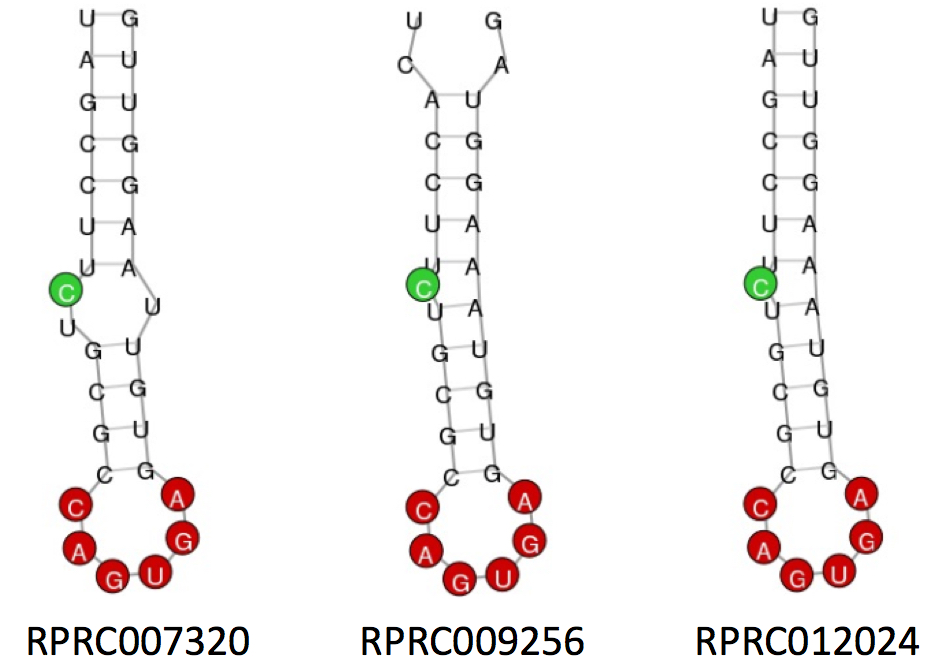


**Supplementary Figure 2: IRE Secondary structures of secreted Ferritin HCH genes.** Prediction of the secondary structure of the IREs used the 5'UTRs of the secreted Ferritin HCH genes. The residues marked in red indicate the canonical type 1 loop. The cytosine base at position 8 (in green) is involved in the interaction with the IRPs. IREs are located in 5 ́UTR of secreted Fer HCH cDNA at the following positions : -119 to -87bp in RPRC007320; -118 to -87bp in RPRC009256 and -120 to -89bp in RPRC012024 from the start codon (AUG) for translation.
