# Supplementary material for: Silencing of Iron and Heme-Related Genes Revealed a Paramount Role of Iron in the Physiology of the Hematophagous Vector Rhodnius prolixus
Source: Front Genet. 2018 Feb 2;9:19. doi: 10.3389/fgene.2018.00019 (PMC5801409; doi:10.3389/fgene.2018.00019)
Supplement: Supplementary file 4 [file Figure_S3.DOCX]

Supplementary Material

SILENCING OF IRON AND HEME-RELATED GENES REVEALED A PARAMOUNT ROLE OF IRON IN THE PHYSIOLOGY OF THE HEMATOPHAGOUS VECTOR *RHODNIUS PROLIXUS*

Ana Beatriz Walter-Nuno, Mabel Taracena Oliva, Rafael D. Mesquita, Pedro L. Oliveira and Gabriela O. Paiva-Silva*


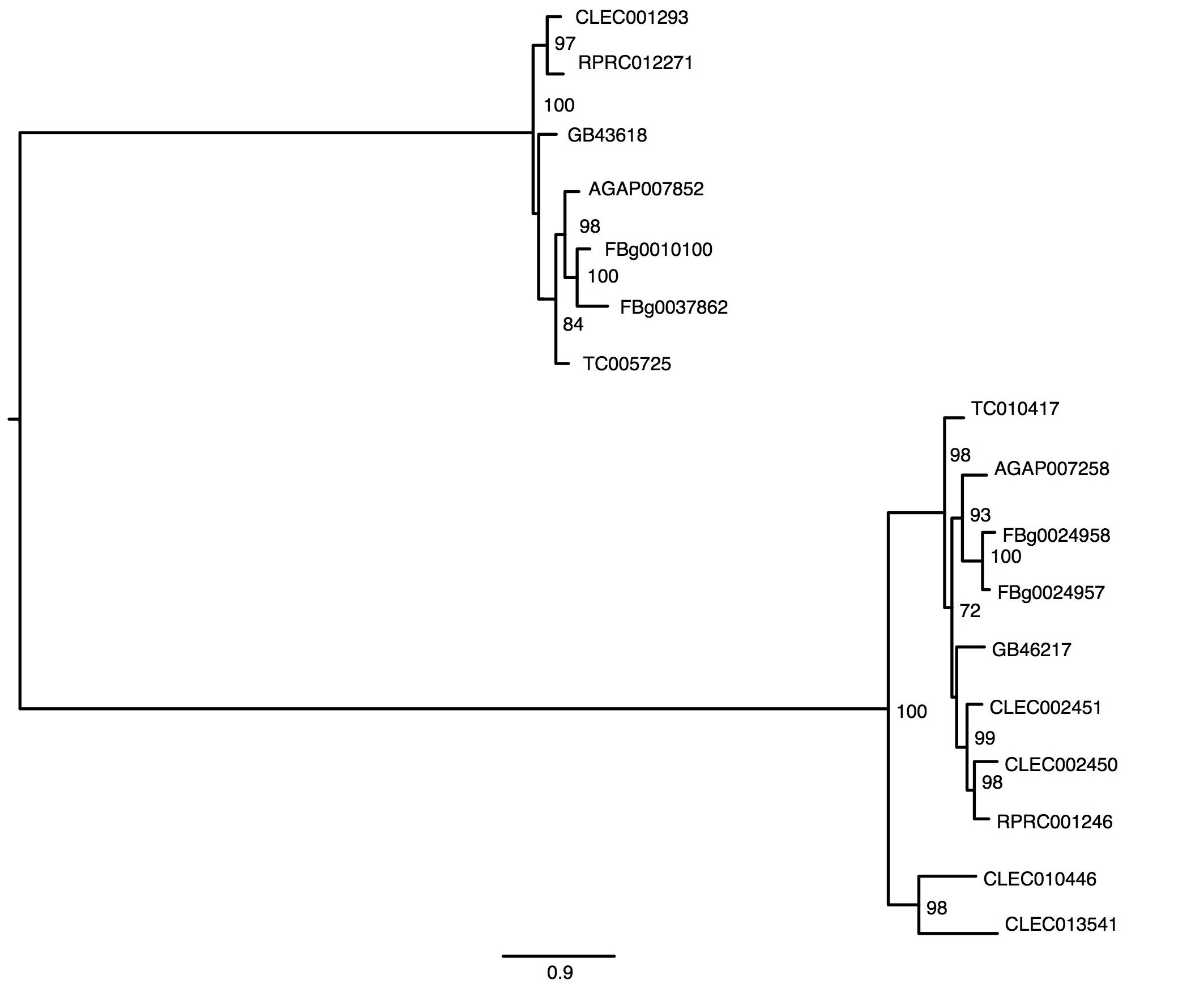


**Supplementary Figure 3**: **Phylogenetic analysis of IRPs insect orthologs.** Maximum-likelihood tree of IRP insect orthologs. Numbers on branches are bootstrap support values from 500 replicates. Only numbers 50% or higher are shown. The sequence codes used were *R. prolixus* (RPRC), *D. melanogaster* (FBg), *T. castaneum* (TC), *C. lectularius* (CLEC), *A. mellifera* (GB) and *A. gambiae* (AGAP).
