# Supplementary material for: Silencing of Iron and Heme-Related Genes Revealed a Paramount Role of Iron in the Physiology of the Hematophagous Vector Rhodnius prolixus
Source: Front Genet. 2018 Feb 2;9:19. doi: 10.3389/fgene.2018.00019 (PMC5801409; doi:10.3389/fgene.2018.00019)
Supplement: Supplementary file 5 [file Figure_S4.DOCX]

Supplementary Material

SILENCING OF IRON AND HEME-RELATED GENES REVEALED A PARAMOUNT ROLE OF IRON IN THE PHYSIOLOGY OF THE HEMATOPHAGOUS VECTOR *RHODNIUS PROLIXUS*

Ana Beatriz Walter-Nuno, Mabel Taracena Oliva, Rafael D. Mesquita, Pedro L. Oliveira and Gabriela O. Paiva-Silva*

*** Correspondence:** Corresponding Author: gosilva@bioqmed.ufrj.br


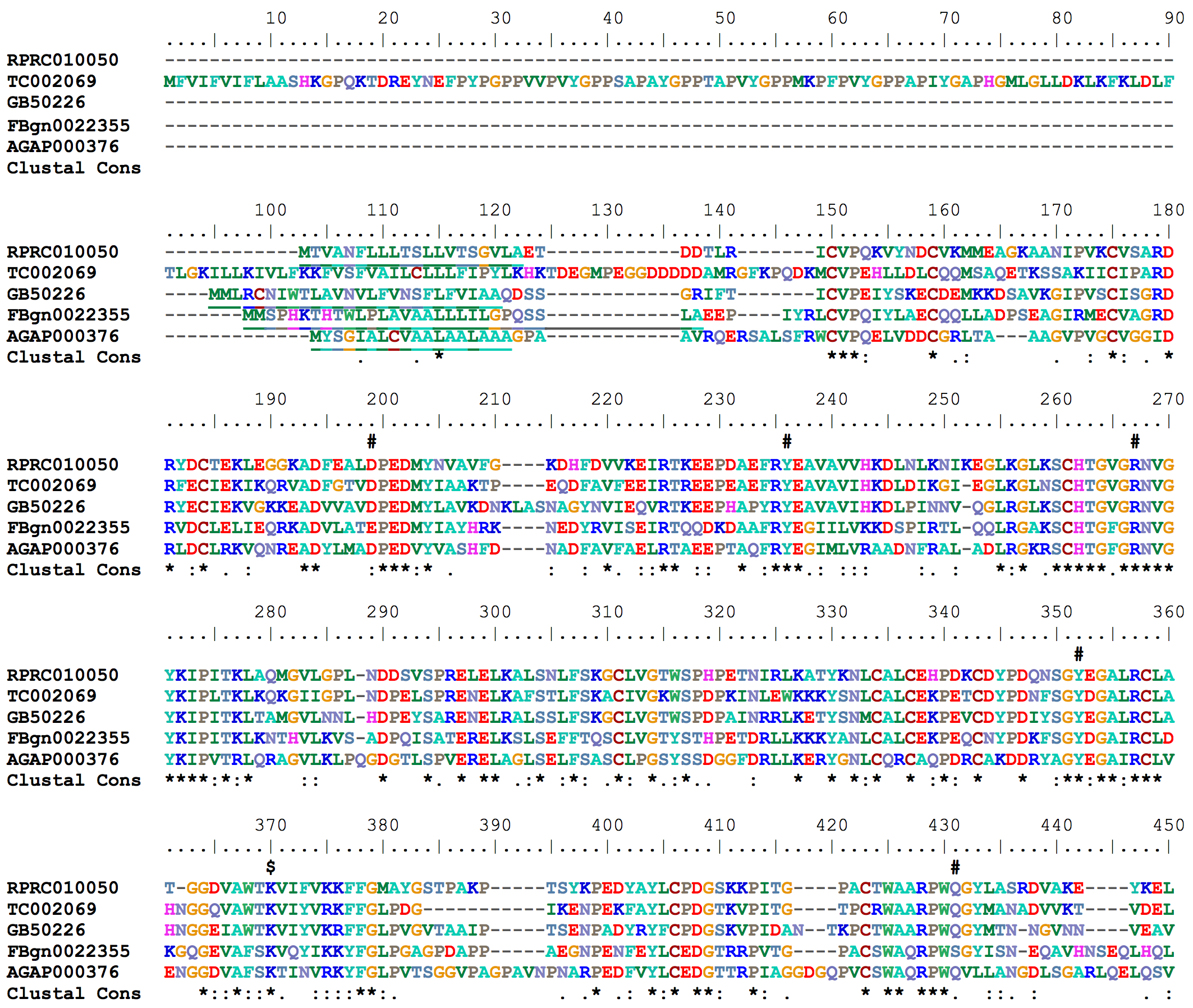


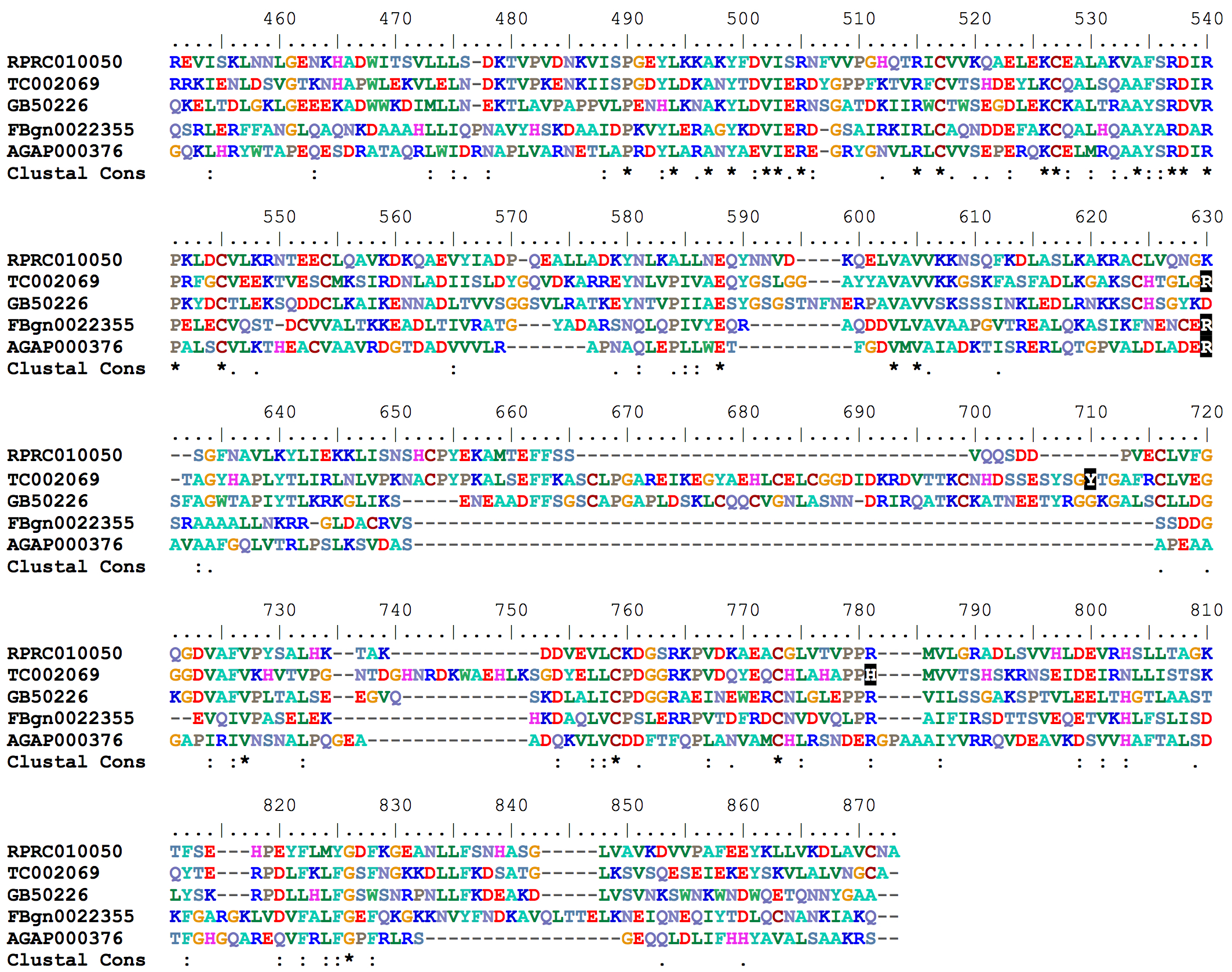


**Supplementary Figure 4:** **Multiple amino acid sequence alignment of *R. prolixus* Transferrin 1 (Tsf-1) with insect orthologs.** Aminoacid color code generated by ClustalW within BioEdit software was used. Consensus information (Clustal cons) was generated by ClustalW. Conserved amino acids are indicated as follows: “#” N-lobe residues involved in iron binding., ”$” Conserved lysine responsible by iron release. C-lobe residues are marked with black background; Signal peptides for secretion are underlined.The sequence ID were *R. prolixus* (RPRC), *D. melanogaster* (FBgn), *T. castaneum* (TC), *C. lectularius* (CLEC), *A. mellifera* (GB) and *A. gambiae* (AGAP).
