# Supplementary material for: Silencing of Iron and Heme-Related Genes Revealed a Paramount Role of Iron in the Physiology of the Hematophagous Vector Rhodnius prolixus
Source: Front Genet. 2018 Feb 2;9:19. doi: 10.3389/fgene.2018.00019 (PMC5801409; doi:10.3389/fgene.2018.00019)
Supplement: Supplementary file 6 [file Figure_S5.DOCX]

Supplementary Material

SILENCING OF IRON AND HEME-RELATED GENES REVEALED A PARAMOUNT ROLE OF IRON IN THE PHYSIOLOGY OF THE HEMATOPHAGOUS VECTOR *RHODNIUS PROLIXUS*

Ana Beatriz Walter-Nuno, Mabel Taracena Oliva, Rafael D. Mesquita, Pedro L. Oliveira and Gabriela O. Paiva-Silva*

*** Correspondence:** Corresponding Author: gosilva@bioqmed.ufrj.br


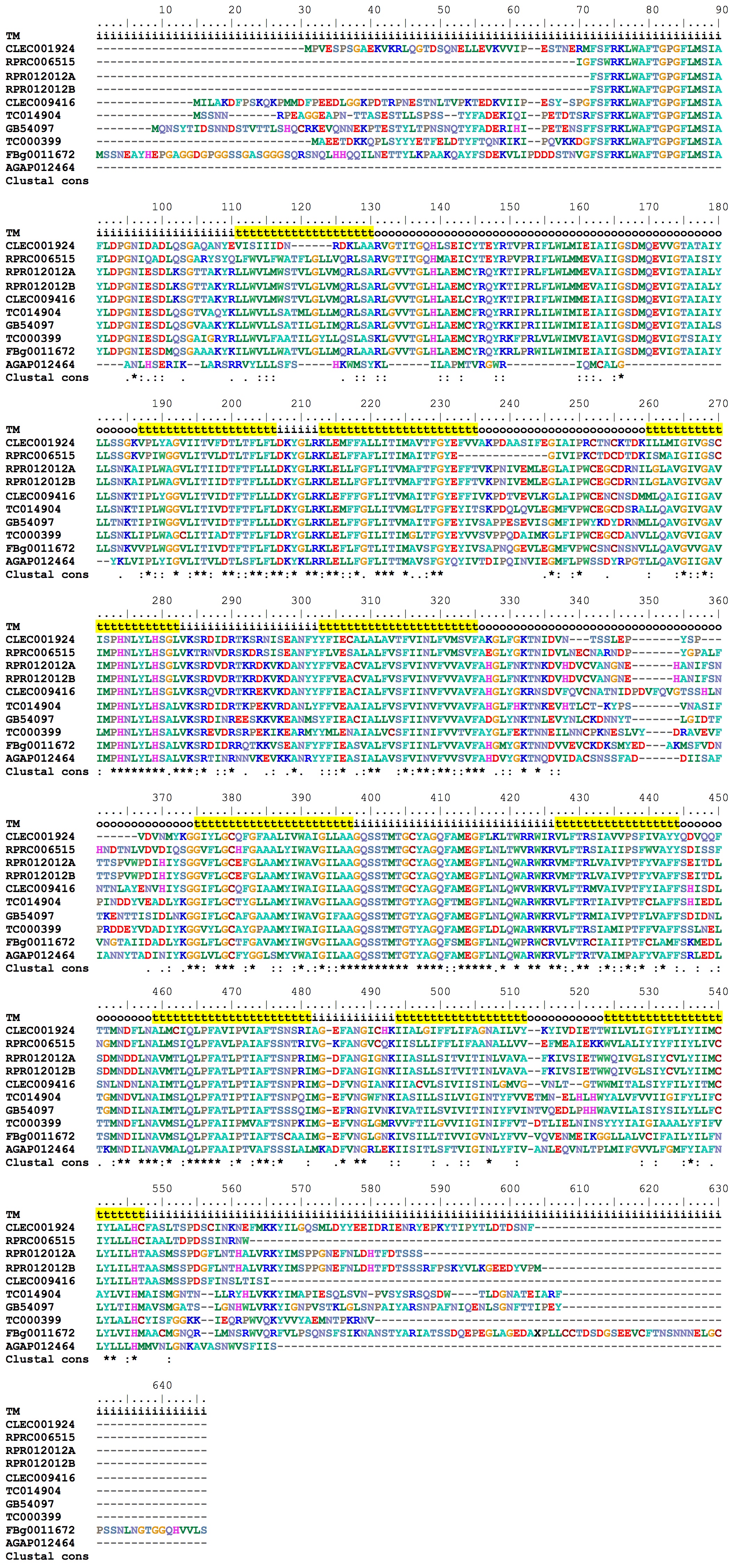


**Supplementary Figure 5: Multiple amino acid sequence alignment of *R. prolixus* Malvolio with insect orthologs**. Aminoacid color code generated by ClustalW within BioEdit software was used.. Consensus information (“Clustal cons” line) was generated by Clustalw. “TM” lines indicate transmembrane regions by yellow background “t”, inside (i) and outside (o) loops. The sequence codes used were *R. prolixus* (RPRC), *D. melanogaster* (FBgn), *T. castaneum* (TC), *C. lectularius* (CLEC), *A. mellifera* (GB) and *A. gambiae* (AGAP).
