# Supplementary material for: Silencing of Iron and Heme-Related Genes Revealed a Paramount Role of Iron in the Physiology of the Hematophagous Vector Rhodnius prolixus
Source: Front Genet. 2018 Feb 2;9:19. doi: 10.3389/fgene.2018.00019 (PMC5801409; doi:10.3389/fgene.2018.00019)
Supplement: Supplementary file 7 [file Figure_S6.DOCX]

Supplementary Material

SILENCING OF IRON AND HEME-RELATED GENES REVEALED A PARAMOUNT ROLE OF IRON IN THE PHYSIOLOGY OF THE HEMATOPHAGOUS VECTOR *RHODNIUS PROLIXUS*

Ana Beatriz Walter-Nuno, Mabel Taracena Oliva, Rafael D. Mesquita, Pedro L. Oliveira and Gabriela O. Paiva-Silva*


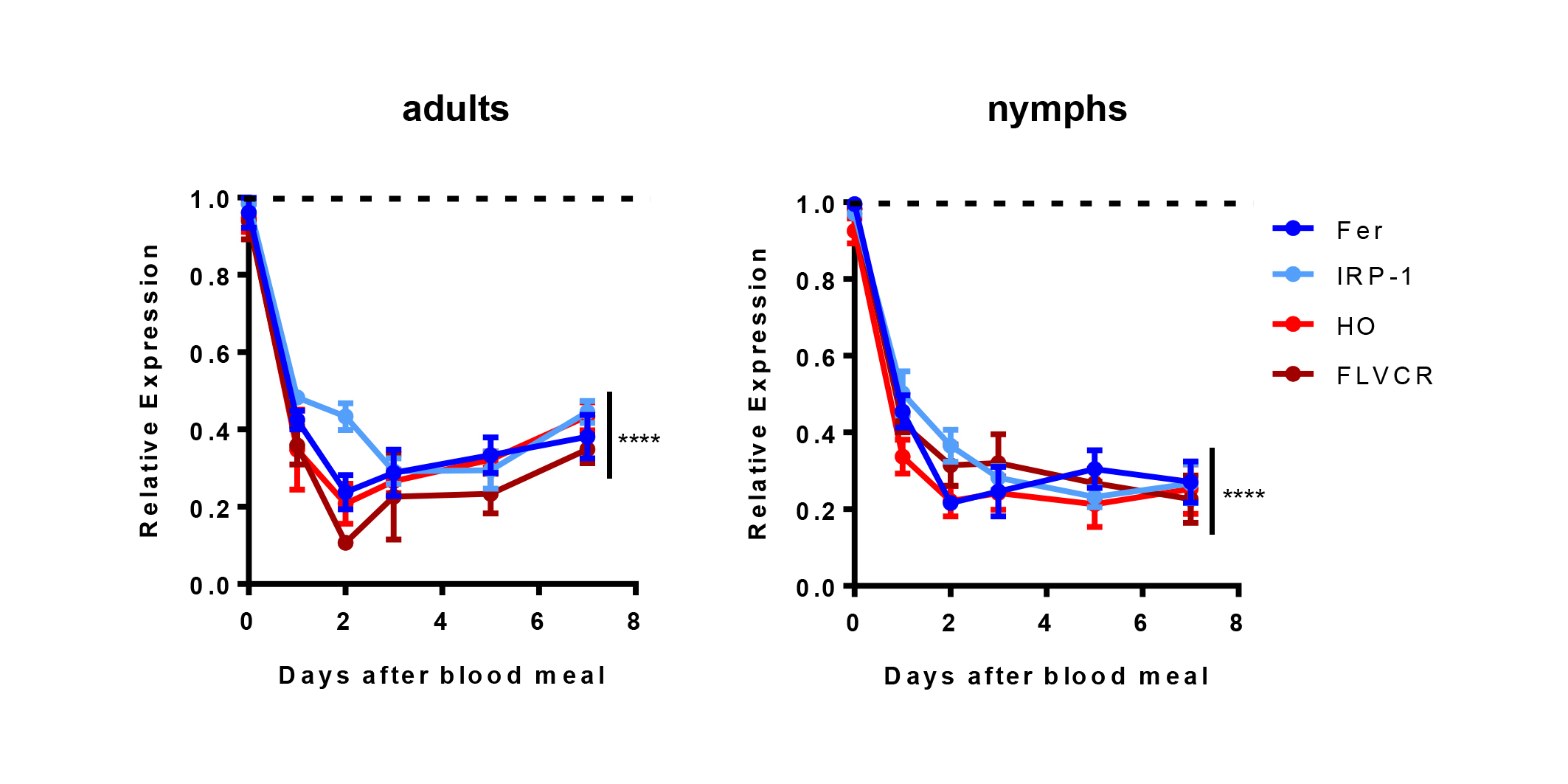


**Supplementary Figure 6:** dsRNA-mediated Knockdown of heme / iron-related genes on days post feeding. Adult females were injected with 1 μg of dsRNA for the following genes Fer; IRP1, HO and FLVCR. Insects were fed on blood 48 hour after dsRNA injection. Nymphs were fed with blood supplemented with dsRNA (1μg/μl) of the same genes. The insects were dissected and the total RNA was extracted from posterior midgut on the different days after feeding. The levels of expression of the genes were determined by real-time PCR. The elongation factor 1 (EF-1) gene was used as an endogenous control. The result was normalized in relation to the animals injected with dsMAL (dashed line). Data on graph is mean ±SE of three independent biological replicates (n=12-15). A two-way ANOVA with Tukey post-test was used to evaluate differences between the expression of the different genes and the control group at different times (**** p<0.0001).
